# Single-cell transcriptomics unveils gene regulatory network plasticity

**DOI:** 10.1186/s13059-019-1713-4

**Published:** 2019-06-04

**Authors:** Giovanni Iacono, Ramon Massoni-Badosa, Holger Heyn

**Affiliations:** 1 CNAG-CRG, Centre for Genomic Regulation (CRG), The Barcelona Institute of Science and Technology, Baldiri Reixac 4, 08028 Barcelona, Spain; 2 Universitat Pompeu Fabra (UPF), Barcelona, Spain

## Abstract

**Background:**

Single-cell RNA sequencing (scRNA-seq) plays a pivotal role in our understanding of cellular heterogeneity. Current analytical workflows are driven by categorizing principles that consider cells as individual entities and classify them into complex taxonomies.

**Results:**

We devise a conceptually different computational framework based on a holistic view, where single-cell datasets are used to infer global, large-scale regulatory networks. We develop correlation metrics that are specifically tailored to single-cell data, and then generate, validate, and interpret single-cell-derived regulatory networks from organs and perturbed systems, such as diabetes and Alzheimer’s disease. Using tools from graph theory, we compute an unbiased quantification of a gene’s biological relevance and accurately pinpoint key players in organ function and drivers of diseases.

**Conclusions:**

Our approach detects multiple latent regulatory changes that are invisible to single-cell workflows based on clustering or differential expression analysis, significantly broadening the biological insights that can be obtained with this leading technology.

**Electronic supplementary material:**

The online version of this article (10.1186/s13059-019-1713-4) contains supplementary material, which is available to authorized users.

## Background

Single-cell RNA sequencing (scRNA-seq) is the leading technology for exploring tissue heterogeneity, unraveling the dynamics of differentiation, and quantifying transcriptional stochasticity. scRNA-seq data are being used to answer increasingly demanding biological questions, which has driven the development in recent years of an array of computational tools for scRNA-seq analysis [1]. Currently, these tools focus on improving features such as clustering, retrieving marker genes, and exploring differentiation trajectories [1]. These scenarios are inspired by a dividing, fragmenting principle, where each cell is an independent identity that must be categorized into different types or stages of increasing hierarchical complexity. This is illustrated by recent large-scale cell atlases that often reach hundreds of stratified (sub)clusters [2]. This has undoubtedly improved our understanding of cell diversity in various biological contexts. However, we hypothesize that a very different approach, inspired by a unifying rather than dividing ideal, would add a novel layer of information that would significantly increase the knowledge gained from single-cell datasets.

Gene expression is tightly regulated by networks of transcription factors, co-factors, and signaling molecules. Understanding these networks is a major goal in modern computational biology, as it will allow us to pinpoint crucial factors that determine phenotype in healthy systems as well as in disease [3, 4]. Unraveling the determinants of a given phenotype provides mechanistic insights into causal dependencies in complex cellular systems. Potentially, single-cell information offers the opportunity to derive a global regulatory network [5]. Traditional approaches to transcriptome profiling, namely microarray and RNA-seq of pooled cells, have been successfully used to infer and characterize regulatory networks, with a recent example using 9435 bulk RNA-seq samples to decode tissue-specific regulatory networks [6]. To date, there are only small-scale efforts to derive regulatory networks from single-cell transcriptomics data, and these efforts have been restricted to specific network properties [7, 8]. This seems unexpected given that single-cell sequencing is the ideal technology for monitoring real interactions between genes in individual cells. However, single-cell data is undermined by a series of technical limitations, such as drop-out events (expressed genes undetected by scRNA-seq) and a high level of noise, which have made it difficult to infer regulatory networks using this type of data [9].

In this paper, we demonstrate the feasibility and value of regulatory network analysis using scRNA-seq datasets. We present a novel correlation metric that can detect gene-to-gene correlations that are otherwise hidden by technical limitations. We apply this new metric to generate global, large-scale regulatory networks for 11 mouse organs [10], for pancreas tissue from healthy individuals and patients with type 2 diabetes [11], and for a mouse model of Alzheimer’s disease [12]. We then validate the resulting networks at multiple levels to confirm the reliability of the reconstruction. Next, we analyze the networks using tools borrowed from graph theory, such as node centralities and dynamical properties. Finally, we integrate network-driven results with standard analyses such as clustering and differential expression analysis and show that key regulators of healthy and diseased systems can only be identified by using integrated, network-based approaches. Together, our results represent the first complete, validated, high-throughput, and disease-centered application of single-cell regulatory network analysis, significantly increasing the knowledge gained from this leading technology.

## Results

### Inferring regulatory networks from large-scale single-cell transcriptomics

**Fig. 1:**
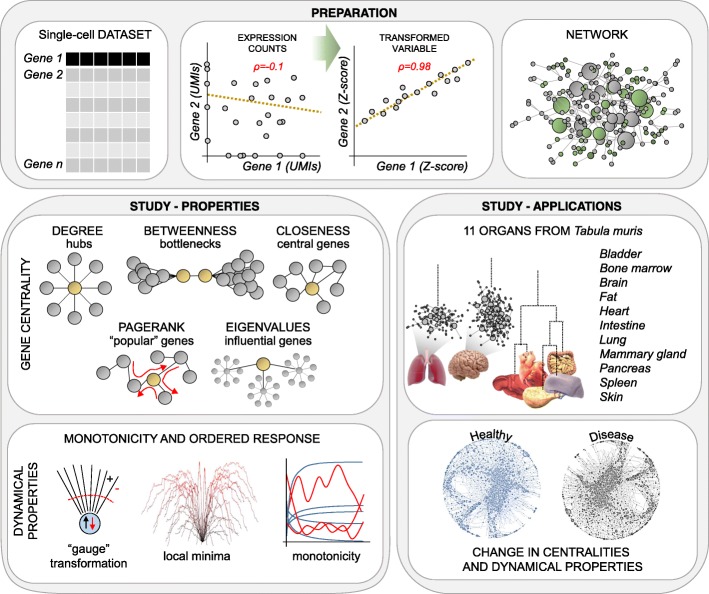
Overview of the computational framework. Preparation: A change of variable (from expression values to *Z*-score) is used to detect otherwise hidden correlations between genes in single-cell datasets, ultimately allowing us to infer the global regulatory network. Gene centrality: Biological importance of genes is quantified using concepts from graph theory. Dynamical properties: We characterize the putative dynamical behavior of the regulatory networks by measuring the monotonicity. Applications: We generated, compared, and characterized the networks of 11 organs in the mouse (Tabula Muris), in the pancreas from healthy and type 2 diabetes human subjects, and in a mouse model of Alzheimer’s disease

**Fig. 2:**
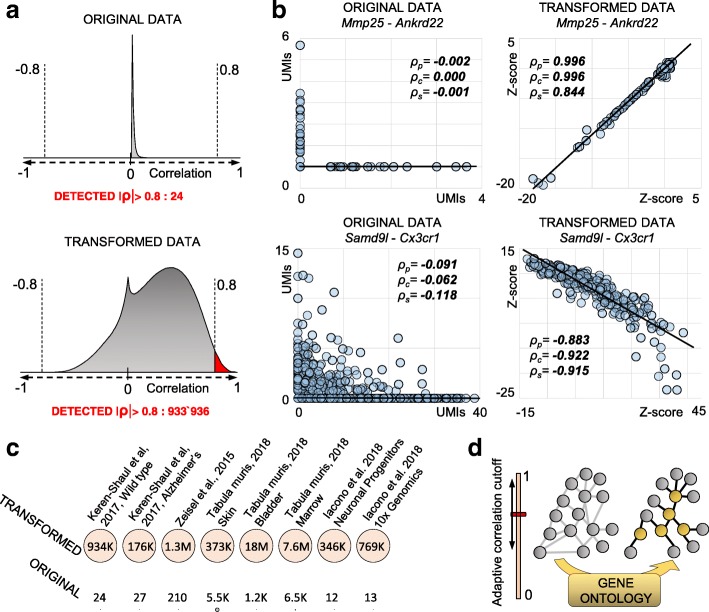
A metric tailored to single-cell data allows detection of hidden correlations. **a** Distribution of *Pearson* correlations *ρ*_p_ in normalized expression data (7697 microglia cells) or in the *Z*-score space. We detect only 24 correlations |*ρ*_p_| > 0.8 in the first scenario, but almost one million |*ρ*_p_| > 0.8 in the *Z*-score space. **b** Examples of correlations using either expression values or *Z*-score-transformed data (*ρ*_p_ *Pearson*, *ρ*_c_ *Cosine*, *ρ*_s_ *Spearman*). Due to drop-out events and other artifacts, the positive correlation between *Mmp25* and *Ankrd22* is only exposed using *Z*-scores. Similarly for the negative correlation between *Samd9l* and *Cx3cr1*. **c** Comparison of detected correlations |*ρ*_p_| > 0.8 using either original expression values or *Z*-score-transformed data across different scRNA-seq technologies, sequencing depths (from 625 [12] to 6480 [13] average detected genes per cell), and source material. **d** An adaptive correlation cutoff and GO annotations are used to infer the regulatory networks from correlation data (see the “Methods” section)

We initially set out to develop a reliable approach for inferring global regulatory networks from single-cell data (Fig. 1). To generate a regulatory network starting from expression data, we require a robust measure of correlation between genes. Unlike in RNA-seq from pools of cells (bulk), single-cell data is inherently noisy and highly sparse, which prevents the effective use of standard coefficients such as *Pearson*, *Spearman*, or *Cosine* correlation, or even *mutual information*. Hence, we conceived a novel correlation measure based on a computational framework tailored to analyze single-cell data, with the rationale that two correlated genes follow similar patterns of differential expression between cell sub-types (see the “Methods” section). Therefore, instead of searching for relationships using the original variables, namely (normalized) expression counts, we compute the correlations between transformed variables, in which expression counts are replaced by *Z*-scores. These *Z*-scores are derived from an unsupervised analysis based on iterative differential expression (DE) between small clusters of cells. To compute *Z*-scores, we exploit a probabilistic model of the noise that considers all sources of variability in single-cell data. Thereby, this approach can detect correlations that would otherwise be concealed by drop-out events and other technical artifacts and, thus, is particularly suitable for single-cell RNA-seq data. When applied to a dataset of 7697 microglia cells [12], we identified 933,936 significant gene-to-gene correlations (*Pearson* > 0.8), a gain of almost 40,000-fold compared to normalized UMI count data (only 24 correlations, Fig. 2a). This large increase in the number of detected correlations is supported by a different distribution in the *Z*-score space compared to the UMIs/reads space (Fig. 2a). Drop-out events can entirely obscure correlations, when genes, although being co-expressed in the same cell type (i.e., cluster), experience mutually exclusive drop-outs (Fig. 2b). When applied to seven additional datasets generated using different scRNA-seq techniques (Fluidigm C1, 10x Genomics Chromium, MARS-seq, Smart-seq2), with different sequencing depths and from different tissue sources [10, 13, 14], the *Z*-score metric consistently outperformed standard approaches, suggesting that it is a valid correlation metric for scRNA-seq data (Fig. 2c).

After identifying gene-to-gene correlations, an adaptive threshold is applied to retain only significant correlations (see the “Methods” section). This adaptivity equalizes the effects of different cell numbers and coverage, and other technical features of scRNA-seq datasets. The retained correlations then become the weighted edges of the regulatory network, with either positive or negative signs. In the final step, gene ontology (GO) information is used to subset the network to “regulators of gene expression,” in order to retain only putative causal (regulatory) relationships (Fig. 2d). Note that using external information (e.g., GO) is an established method for refining networks [15–17]. To determine the importance of a given gene in a single-cell regulatory network and its underlying biological system, we applied analytical tools from the field of graph theory. These tools allow us to quantify the biological relevance of a gene using various measures of centrality, namely *degree*, *betweenness*, *closeness*, *pagerank*, and *eigenvalues* (Fig. 1). For example, genes with a high *betweenness* centrality are crucial for the flow of information between network modules (bottlenecks), genes with a high *closeness* can rapidly spread a signal across the network, and genes with a high *eigenvalue* are highly influential (see the “Methods” section).

### Benchmarking inferred correlations

**Fig. 3:**
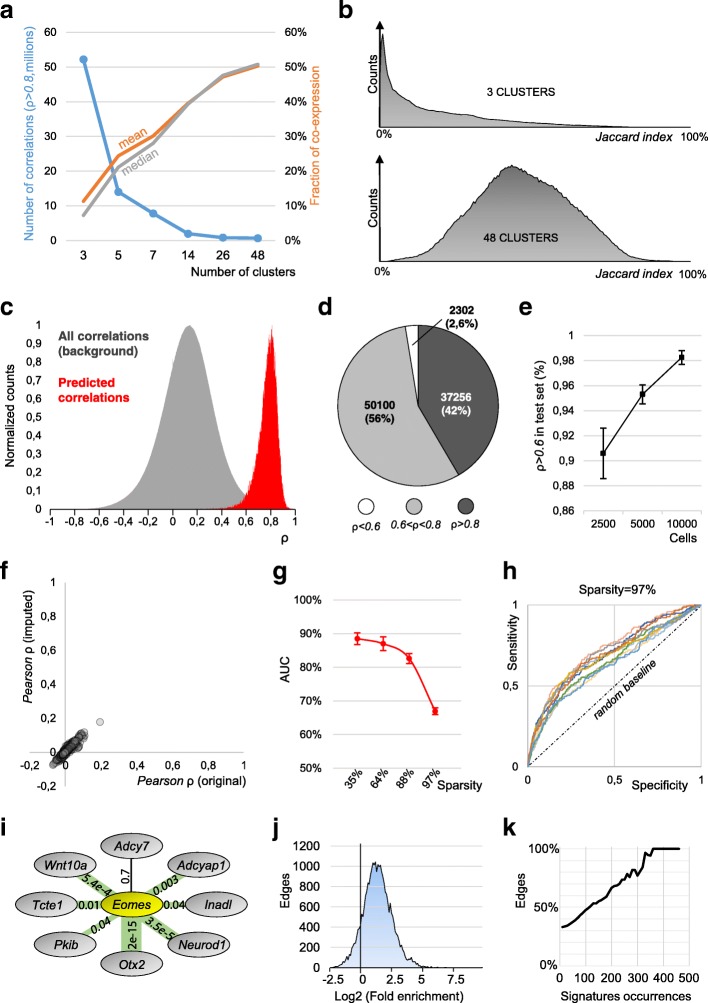
Technical benchmarks. **a** Left axis, amounts of detected correlations (sensitivity) decrease with the number of clusters. Right axis, mean and median of co-expression (specificity) increase with the number of clusters. **b** Distribution plots of the co-expression (Jaccard index) for 3 (top) and 48 (bottom) clusters. **c** Distribution plots showing all the correlations of the testing dataset (gray) compared with a selected subset (those predicted by the training dataset). **d** Pie chart, composition of the predicted correlations in the testing dataset (5000 cells). **e** Relationship between size of the set and resulting predicted correlations with *ρ* > 0.6 (mean ± S.E.M.). **f** Scatter plot of correlations inferred from the original counts against those inferred from imputed counts (scImpute). The two distributions are correlated with *Pearson ρ* = 0.983. **g** AUC of inferred correlations, simulated data, 10 independent repetitions for each sparsity (error bars S.E.M.). **h** ROC curves for 10 simulations with the highest sparsity of 97% are above the random ROC line. **i** Example of the validation of *Eomes* neighbors in the brain network. For each edge, we computed fold enrichment (proportional to edge width, highest *Otx2* with 9.87, lowest *Adcy7* with 0.83) and a *p* value (labels). In the case of *Eomes*, all edges but one (*Adcy7*) are validated with *p* < 0.05. **j** Overall distribution of edge-wise fold enrichment in the brain network was biased towards positive values, suggesting that neighboring genes are regulated together in perturbed systems. **k** Brain network, percentage of validated edges with *p* < 0.05 (*y*-axis) filtered by the number of MSigDB occurrences (*x*-axis). Higher percentages of validated (*p* < 0.05) edges are obtained by considering only edges with high occurrences

The inferred correlations were linearly proportional to the correlations computed over the average gene expression per cluster, confirming that the approach is not introducing global biases nor artifacts (Additional file 1: Figure S1a). The quality of the inferred correlations critically depends on the number of clusters: a tradeoff between sensitivity and specificity. Partitioning cells in few, large clusters allows a sensitive DE analysis, however, with reduced specificity. For example, partitioning 3005 brain cells [14] in three clusters detected 52 million correlations (*ρ* > 0.8, Fig. 3a). However, the median co-expression of correlated gene pairs was only 7% (Jaccard index), indicating that most of the correlated genes are not expressed in the same cell (Fig. 3a, b). As three clusters are not sufficient to segregate sub-types, cells with different phenotypes enter the same cluster and generate false-positive correlations. Importantly, our approach applies a recursive clustering that maximizes the number of biologically informative clusters, increases the quality of correlations, and preserves sensitivity (see the “Methods” section). Exemplarily, recursive clustering divides the 3005 brain cells into 48 clusters, increasing the average co-expression to 51% (Fig. 3a, b). Of note, drop-out events impede the detection of a complete co-expression of 100%*.*

Next, we quantified the false discovery rate (FDR) of the inferred correlations using the 3005 brain cells and random cluster assignment as the null model. Any correlation found with the random clustering must be false positive, originated from an uncontrolled sensitivity in the DE analysis and subsequent *Z*-score-based correlation. We applied a reshuffling of the 48 clusters to generate random assignments without altering the distribution of cluster sizes. We observed an average FDR of 0.062 ± 0.012%, indicating that the approach has a low detection of false-positive events (S.E.M., 5 independent repetitions of random clusters; Additional file 1: Figure S1b).

We then tested if the approach was a robust predictor of correlations. To this end, we randomly sampled two non-overlapping groups of 5000 cells from the 1.3 million dataset of mouse brain cells [18], to quantify the extent to which correlations inferred from a training set (group 1) can predict correlations in a test set (group 2). Correlating genes in the training set were very likely to be correlated in the testing dataset, as illustrated by a clear distribution shift compared to the background (approximate *p* value < 1e−31128, Wilcoxon signed-rank test; Fig. 3c). Specifically, 42% (97.6%) of correlations found in the training set (*ρ* > 0.8) revealed correlations of *ρ* > 0.8 (*ρ* > 0.6) in the test set (Fig. 3d). We repeated this simulation for smaller (2500 cells) and larger (10,000 cells) datasets, consistently determining the inferred correlations as valid predictor (Fig. 3e). Overall, these results indicate that our inferred correlations are reproducible, with datasets of similar type (e.g., biological replicates) yielding similar correlations. Importantly, the robustness of the prediction increased with the dataset size, suggesting that the approach efficiently exploited higher cell numbers to infer more robust correlations. This is particularly important considering the trend towards very large datasets in single-cell transcriptomics studies [19].

Imputation is utilized in single-cell dataset to replace drop-out events with non-zero estimates of predicted expression values. We assessed if imputation improves correlation coefficients of transcript count data and therefore constitutes a viable alternative to the here presented approach. We applied scImpute [20], a widely used and benchmarked imputation tool [21, 22], on 8333 microglia cells [12], an extremely sparse and challenging dataset (6.1% non-zero values after filtering for expressed genes). Although imputation was able to decrease the sparsity (non-zeros increased to 20.4%), the improvement was not sufficient to detect correlations (*Pearson ρ* > 0.8, Fig. 3f). We further tested MAGIC, a Markov affinity-based graph imputation method, previously shown to be able to denoise count matrixes and to fill in missing transcripts [23]. The imputed dataset was able to detect large amounts of correlations (*Pearson* coefficients *ρ* > 0.8), however, with excessive amounts of false-positive correlations compared to our approach (Additional file 1: Figure S1c), a common artifact of imputation methods [24]. In line with previous observations [24, 25], we concluded that imputation, although certainly valuable in aiding clustering and phenotyping, is not sufficient to cope with the detrimental effects of drop-out events, preventing correlation analysis of single-cell expression count data.

We next sought to further validate our approach using simulations [26]. First, we simulated a single-cell “reference dataset” with minimal sparsity (3% sparsity, 97% non-zero values), which was used to calculate the true positive correlations. Next, we generated testing datasets by adding increasing levels of drop-out events (35%, 64%, 88%, and 97% sparsity). The performance of our approach in recovering true positive correlations was then quantified with the AUC (area under the curve) ROC (receiver operating characteristics) curve. Our approach performed optimally up to the sparsity of 88% (AUC = 83%, Fig. 3g) and still performed better than random chance at the highest sparsity (97%, AUC = 67%, Fig. 3g, h).

In principle, segregating a dataset with recursive clustering and computing average gene expression per cluster might sufficiently mitigate the effects of drop-out events and improve the performance of correlation metrics. However, averaged expression values were considerably more skewed than *Z*-scores, presenting few outliers with high expression levels (Additional file 1: Figure S1d, f). As a result, the correlations inferred by cluster-average expression values are mainly driven by outliers (highly positive clusters). In contrary, *Z*-scores use the information of all clusters, making them overall more accurate (Additional file 1: Figure S1d, e, f).

In summary, the benchmarking confirmed our approach for inferring correlations to be accurate, scalable, robust, artifact-free, and out-performing imputation-based approaches.

### Single-cell regulatory networks identify essential and specific genes for organ function

To evaluate the value of using large-scale regulatory networks inferred from single cells to aid biological interpretation of scRNA-seq datasets, we first applied our framework to a single-cell resolved mouse organ atlas [10]. We generated regulatory networks from 11 organs: endoderm (lung, pancreas, intestine), mesoderm (heart, fat, spleen, bladder, bone marrow), and ectoderm (skin, brain, mammary glands). The adaptive correlation threshold, required to normalize batch effects such as sequencing depth or cell numbers, reached high values for all organs (*ρ*_thresh_ > 0.9, Table 1), which confirms the significance of selected correlations. Inferred networks had a scale-free topology (a structure conferring fault-tolerant behavior, *p* > 0.01 Kolmogorov–Smirnov test, Additional file 1: Figure S2) which is in line with previous findings in manually curated networks [27–29].

**Table 1:** Overview of specifications for inferred regulatory networks. In order: the adaptive correlation threshold set to retain significant correlations; average detected genes, number of edges, and percentage of negative edges; number of nodes and percentage of nodes being “regulators of transcription”; network density (ratio edges/nodes), number of connected components, average shortest path, and modularity

Network	Corr. Tresh.	Avg. det. genes	Edges	Edges < 0 (%)	Nodes	RT %	E/N	Conn. Comp.	Avg. short. path	Modularity
Marrow	0.95	3152	48,748	0.0	3221	27.5	15.13	3	4.06	0.37
Intestine	0.94	4046	60,941	0.0	5858	24.4	10.40	1	6.33	0.49
Heart	0.94	2682	3460	0.4	1519	23.7	2.28	3	6.71	0.75
Brain	0.92	3195	23,492	0.0	5131	27.3	4.58	2	9.84	0.73
Fat	0.89	3409	35,495	1.4	4814	26.7	7.37	3	5.27	0.42
Mammary	0.95	3623	38,088	1.2	5978	26.0	6.37	2	7.92	0.64
Skin	0.85	3410	42,965	8.6	5361	28.6	8.01	1	5.56	0.59
Spleen	0.88	1839	30,424	3.4	4560	28.8	6.67	1	6.95	0.43
Pancreas	0.92	4408	30,191	0.5	6586	24.6	4.58	1	8.84	0.75
Bladder	0.99	4873	46,136	26.2	5729	22.8	8.05	5	7.19	0.52
Lung	0.90	2546	15,008	0.6	2921	25.7	5.14	2	7.73	0.66
Pancreas, healthy	0.90	5580	48,540	1.1	7432	28.0	6.53	2	7.06	0.58
Pancreas, T2D	0.91	6636	52,035	6.7	7501	27.0	6.94	1	6.63	0.57
Microglia, healthy	0.89	625	32,064	0.0	2665	31.6	12.03	3	4.16	0.45
Microglia, AD	0.84	676	20,970	0.0	2774	26.7	7.56	1	6.74	0.65

As expected, the number of nodes (i.e., genes) in the network scaled with the average number of detected genes (*Pearson ρ* = 0.82, Table 1). All networks had a positive modularity, indicating a structure organized into multiple, separated modules of genes (also called communities). The most modular networks were the pancreas, heart, and brain, and the bone marrow was the least modular (Table 1). Interestingly, networks showed a wide variation in their density (ratio edges/nodes). Lower network densities could indicate a frequent use of “indirect” transcriptional regulation, signaling cascade involving genes without direct gene regulatory function (see the “Methods” section). Of note, network density and modularity showed an inverse relationship (*Pearson ρ* = − 0.8, Table 1), suggesting that sparser networks (such as the brain, heart, and pancreas) preserve a strong intra-modular connectivity at the expense of decreased inter-modular connectivity (Table 1). Hence, modularity represents a proxy for the tissue heterogeneity, with an increased phenotype diversity being related to more modular networks.

We next sought to validate our predicted regulatory edges. We reasoned that when a system is perturbed, pairs of connected genes (linked by an undirected edge), on average, are more likely to be activated/deactivated together. Thus, we used the Molecular Signature database (MSigDB), which contains an extensive collection of experimental signatures representing perturbations in different biological systems. We performed a proportional test (*Fisher’s* exact test) to quantify the co-occurrence of neighboring genes in MSigDB experimental signatures, thereby testing the significance of each individual edge in the network (Fig. 3i). In the brain network, the edges (23,492) showed an overall distribution bias towards positive fold enrichment and significant *p* values, which supports our inferred regulatory links (Fig. 3j, k). Specifically, 34% of the edges were validated (*p* < 0.05), and this percentage increased when we considered only the edges whose genes are present in many MSigDB signatures (Fig. 3k). In fact, 100% of the edges were validated when considering only genes appearing in at least 360 signatures. The results were similar for the other 10 mouse organ networks (Additional file 1: Figure S3a).

**Fig. 4:**
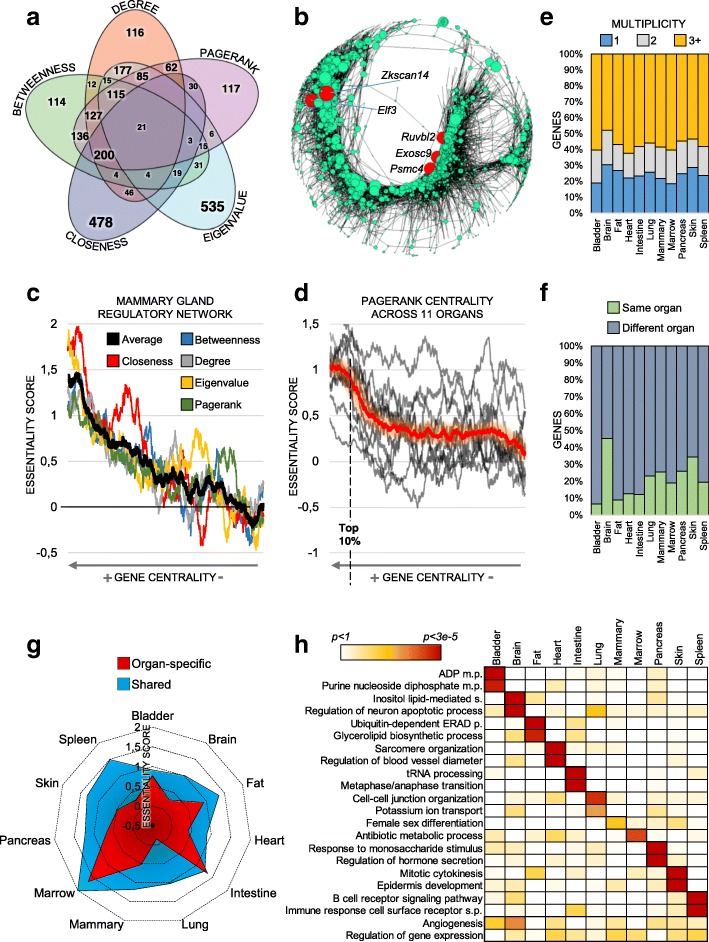
Regulatory networks inferred from 11 organs of the mouse body. **a** Marginal overlap of genes of different centralities in the brain network (top 20% genes). Additional organs in Additional file 1: Figure S5. **b** Visual representation of the mammary gland network, in which node size is proportional to *pagerank* centrality. The top five central nodes (red color) are all genes classified by OGEE as biologically essential. **c** Genes (2403) of the mammary gland regulatory network sorted by centrality. Increasing centrality corresponds to higher biological essentiality for all centrality measures. **d** Genes sorted according to their *pagerank* centrality. High centrality (roughly top 10% of genes) corresponds to high biological essentiality. **e** The central genes (here, *pagerank*, top 20%) of each organ classified by their multiplicity. Multiplicity = 1 means that they are central only in that organ, whereas multiplicity = 2(3+) means that they are also central in additional organs (total of 2, or 3 or more, organs). Additional organs in Additional file 1: Figure S3B. **f** Analysis of genes which are (i) central in at least one organ (*pagerank*) and (ii) upregulated in one organ compared to others. Intriguingly, most of the genes central in a given organ are actually expressed to a significantly higher extent (*p* < 0.05) in a different organ. Additional organs in Additional file 1: Figure S3D. **g** Radar plot of the *pagerank* ES score for the central genes of each organ (top 20%), partitioned in either organ-specific or shared (multiplicity 3+). The latter have higher biological essentiality and reach a significant *p* value for all organs (random permutations, *p* < 0.005 Additional file 1: Figure S3C). **h** Heatmap of GO enrichments using *degree* centrality

Notably, genes that were central in the different measures showed marginal overlap (Fig. 4a, Additional file 1: Figures S4, S5), which suggests that conceptually different centralities quantify distinct types of biological importance and provide mutually complementary information.

To confirm the importance of central regulatory genes in the biological system, we calculated their enrichment among experimentally validated essential genes (Online GEne Essentiality (OGEE) database); knockdown of these genes causes lethal or infertile phenotypes in *Mus musculus* (see the “Methods” section). For all centrality metrics, gene centrality was proportional to biological essentiality (Fig. 4b, c), which supports the reliability of our networks and the validity of applying node centrality theories to single-cell data. These results also suggest that, in principle, all the tested centralities yield biological insights. However, some centralities (*pagerank*, *betweenness*, *degree*) produced more stable predictions irrespective of the network structure (Fig. 4d, Additional file 1: Figure S6). For example, *closeness* centrality did not perform well on disconnected graphs.

Next, to assess genes’ organ-specific centrality and how this relates to biological functions, we compared the centrality of genes across organs. In 11 regulatory networks, we identified genes that were central for single or multiple mouse organs (Fig. 4e, Additional file 1: Figure S3b). Genes that were central in multiple organs appeared more essential than organ-specific genes (Fig. 4g, Additional file 1: Figure S3c). In line with this, shared central genes were associated with general housekeeping functions (e.g., gene expression or metabolic processes), whereas organ-specific central genes were associated with organ-specific processes (GO enrichment, Fig. 4h). Examples include *epidermis development* in the skin (*p* < 3.2 × 10^−5^), *regulation of blood vessel diameter* in the heart (*p* < 4.1 × 10^−5^), and *regulation of neuron apoptotic process* in the brain (*p* < 5.8 × 10^−4^). Importantly, regulatory network analysis provided biologically relevant information not captured by gene expression levels alone. In fact, most organ-specific central genes are not upregulated in their respective organs (Fig. 4f). This implies that gene expression levels are not an adequate measure of the importance of such genes for their underlying biological system.

Overall, our framework for single-cell network analysis was capable of exposing functional regulatory structures and key genes that are undetectable by current computational strategies. We believe that this approach will be very valuable and broadly applicable for interpreting healthy and diseased complex biological systems. For the latter, regulatory networks will allow us to detect the molecular fingerprints of perturbations and to identify key driver genes for disease.

### Altered regulatory network architecture in the pancreas from type 2 diabetes (T2D) patients

**Fig. 5:**
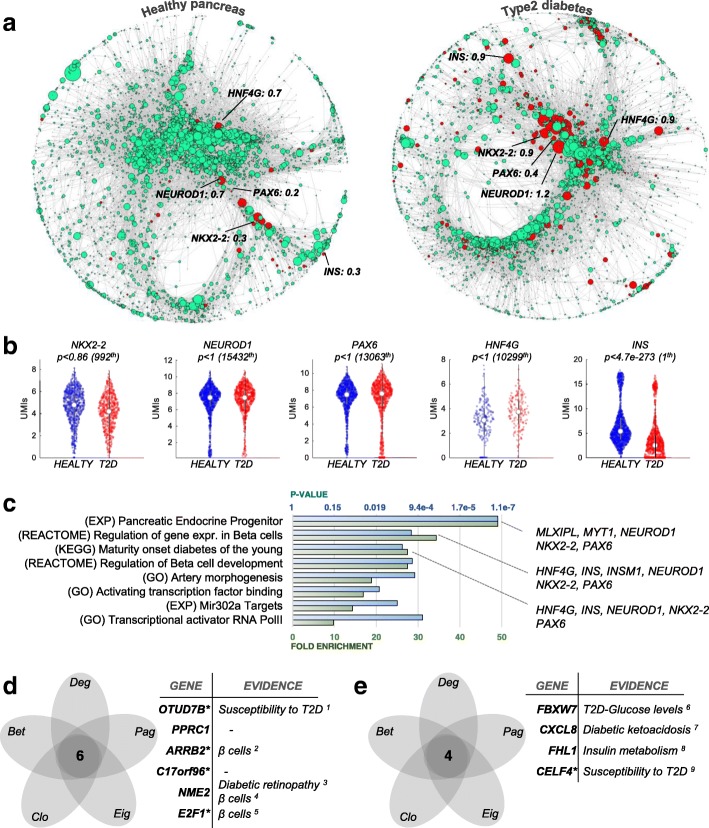
Changes in centralities and dynamical properties in the pancreas of type 2 diabetes (T2D). **a** Network from healthy and T2D subjects, in which node size is proportional to its *pagerank* centrality. In red are the nodes which are present in both networks and have higher *pagerank* in T2D (84 nodes). **b** Violin plots, *p* values, and ranking in differential expression in healthy vs. T2D tissue using all cells (1313 control cells vs 1178 T2D cells). **c** GO and MSigDB enrichments showing overrepresentation of diabetes-related functions in nodes with increased *pagerank*. **d**, **e** Several genes showing simultaneous decrease (**d**) or increase (**e**) of the five centralities have been implicated in diabetes

We considered that regulatory networks and gene centralities would be particularly informative about latent disease-related regulatory changes that are invisible to current analytical approaches. Thus, we generated healthy and T2D regulatory networks for 2491 single-cell transcriptomes from diabetes patients and controls [11]. First, we studied disease-related changes in *pagerank* centrality, a metric originally conceived to rank the popularity of websites. Nodes with high *pagerank* centrality indicate “popular” genes involved in multiple regulatory pathways. We hypothesized that genes with altered *pagerank* centrality would represent T2D regulatory changes with high functional impact on disease pathology. We found 162 genes with significantly increased *pagerank* centrality in T2D, despite showing equal expression levels (*p* > 0.05) in T2D patients and healthy controls (Fig. 5a, b). In addition, we detected 10 genes, including *insulin* (*INS*), with increased *pagerank* that were significantly downregulated in T2D (*p* < 0.05). Consistent with known disease pathology, *insulin* was the most downregulated gene (*p* < 2.2 × 10^−264^), but had significantly higher *pagerank* centrality (from 0.3 to 0.9; Fig. 5a, b). This shows that insulin is a crucial limiting factor in the T2D network, and further emphasizes its pivotal role for the disease. Next, we used GO and MSigDB to confirm the importance of the 172 genes with increased *pagerank* for pancreas function. Gene set enrichment supported their role in diabetes pathophysiology, as illustrated by the overrepresentation of terms such as “onset of diabetes in the young” signature (*p* < 0.016; Fig. 5c). Genes showing changes in the remaining centralities (*eigenvalues*, *closeness*, *betweenness*, *degree*) were also enriched in diabetes-related functions, further highlighting the value of our method for interpreting scRNA-seq data (Additional file 2).

Finally, we identified genes with a simultaneous increase or decrease in the five centralities, which we expect to drive essential regulatory changes in T2D. We detected 4 (6) genes with repeated increased (decreased) centrality, most of which have previously been linked to diabetes pathology (Fig. 5d, e) [30–37]. For example, *ARRB2*, a gene with a demonstrated role in *β* cell development [38], showed no differential expression (*p* > 0.05) but was simultaneously decreased in all six centrality measures. This is particularly remarkable because *β* cells were the most deregulated cell type in the original analysis [11], which however did not detect the importance of *ARRB2* in this context. This further supports the notion that generating global regulatory networks from single-cell data provides important insights into the pathological mechanisms of diseases. We further measured the degree of monotonicity to test whether the T2D network had a more chaotic dynamical behavior compared to the healthy pancreas network, but did not find significant changes for this attribute (Additional file 1: Figure S7c, d).

### Network-driven interpretation of differentially expressed genes

**Fig. 6:**
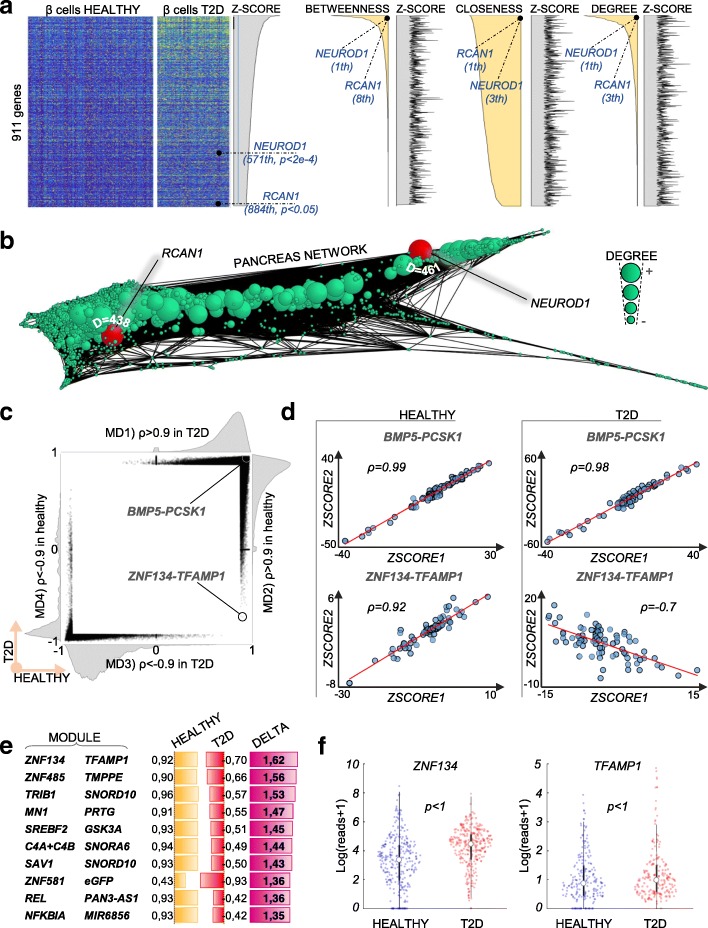
Prediction of gene importance in DE data and directional changes in correlations. **a** Heatmap of normalized expression values of 911 genes found significantly upregulated in T2D β cells compared to healthy β cells (*p* < 0.05) sorted by decreasing *Z*-score (i.e., increasing *p* values) or decreasing centralities (*betweenness*, *closeness*, and *degree*). Biological importance of *NEUROD1* and *RCAN1* is highlighted by their high centrality but not by their DE *Z*-scores. In general, correlation between DE *Z*-scores and centrality is marginal, as shown by the erratic area plots of *Z*-scores sorted by centrality. **b** Pancreas regulatory network generated using all cells (both healthy and T2D), node size proportional to its degree. *NEUROD1* and *RCAN1* have high *degree* centrality (461 and 438 respectively). **c** Scatter plot and marginal distributions (MD1–4) of all pairwise gene correlations with |*ρ*| > 0.9 (329,046 couples). The strong bias in the marginal distributions (especially MD1, 2, 4) indicates an overall similarity of correlations between healthy and T2D pancreas. **d** An example of a highly conserved correlation (*BMP5-PCSK1*) against the strongest inversion (*ZNF134-TFAMP1*). **e** Overview of top 10 inversions of correlations. **f** Neither ZNF134 nor TFAMP1 display a significant change of expression (DE)

Differential expression (DE) is the backbone of most analytical pipelines for RNA-seq. A typical challenge is to interpret differentially expressed genes and identify functionally important events. This is generally achieved by (i) focusing on the genes with most significant *p* value, (ii) integrating external databases (GO or MSigDB) to elucidate key genes and pathways, or (iii) using personal knowledge to identify previously annotated genes. However, none of these approaches guarantees an unbiased classification of biological importance. In fact, in DE analysis, *p* values rank genes by technical reproducibility, not by biological importance, and both external databases and personal knowledge can be biased. Single-cell regulatory networks can be used to provide an unbiased, hypothesis-free classification of the biological importance of genes, allowing us to automatically identify pivotal deregulated genes, which greatly facilitates data interpretation. Comparing gene expression in *β* cells between healthy and T2D individuals, we detected 911 genes upregulated in T2D *β* cells (*p* < 0.05; Fig. 6a). Ranking these genes by centrality rather than *p* values (i.e., *Z*-scores) provided quantitative sorting by biological importance, allowing us to immediately focus on the most relevant candidates. For example, *NEUROD1* and *RCAN1* showed the highest centrality of all deregulated genes according to multiple metrics (Fig. 6a, b), suggesting that they are the most informative and biologically relevant. Interestingly, mutations in *NEUROD1* were associated with T2D [39], whereas upregulation of *RCAN1* was shown to cause hyperinsulinemia, *β* cell dysfunction, and diabetes [40]. Notably, neither of these genes was highlighted with DE *p* values (*NEUROD1* 2829th, *p* < 2 × 10^−4^; *RCAN1* 4331th, *p* < 0.05). This example highlights the high additive value of using single-cell regulatory networks and related node centralities to aid interpretation of DE results.

### Inversions of gene correlations in T2D

Regulatory networks can be further interrogated to detect changes in local interactions, namely pairwise correlations between genes. We reasoned that gene pairs with an inverted correlation in T2D samples compared to the healthy pancreas represent rewired functional modules with potential pathological implications. Of note, a complete inversion of correlation, from positive (training set, *ρ* > 0.9) to negative (test set, *ρ* < 0), never occurred when benchmarking the correlation approach (25,632 computed cases, *p* < 1/25,632; Fig. 3c–e). Performing comparative analysis of the healthy and T2D networks, all pairwise correlations were highly similar under healthy and T2D conditions (example of *BMP5* and *PCSK1*, Fig. 6c, d), which is remarkable considering that the data come from different donors and are subject to inter-individual variability as well as several confounding factors (e.g., age and weight). In contrary, correlations inferred directly from expression data were few, in line with our previous results on other datasets (Fig. 2c), and unreproducible across conditions (Additional file 1: Figure S7a, b). This indicates that our approach works transversely to confounding variables, ultimately exposing the true functional correlations between genes (see the “Confounding variables” section in the “Methods” section). Closer inspection revealed a number of modules (14) with strongly (*ρ* > 1) inverted correlations, the most striking example being *ZNF134* and *TFAMP1*, which switch from a strong positive correlation in the healthy pancreas (*ρ* = 0.92) to a negative correlation in T2D (*ρ* = − 0.7). Neither of these genes showed a change in expression between conditions (healthy/T2D), which renders their altered functionality invisible to standard methods (Fig. 6e, f).

Several other genes displaying inverted correlations have previously been linked to diabetes (7/18), either by functional studies (*TRIB1*, glucose metabolism; *NFKBIA*, insulin resistance pathway) or as candidate disease genes in GWAS or gene expression studies (*TMPPE*, *PRTG*, and *ZNF319*) [41–46]. Functionally, the most interesting are *SREBP2* and *GSK3A*, which have a direct mechanistic relationship and are both implicated in T2D and which also switched from a positive to a negative correlation. SREBP transcription factors are major players in lipid metabolism and possibly insulin resistance, whereas GSK3 phosphorylates SREBP in the absence of insulin and AKT signaling, leading to its degradation [47–49]. Consequently, we can speculate that the reversal in correlations inferred from single-cell data is directly related to a change in insulin signaling and the degradation of SREBP2 through GSK3A*.*

In summary, the comparative analysis of single-cell-driven correlations is a suitable novel approach for disentangling the molecular mechanisms of diabetes and further enlarges the repertoire of single-cell data analysis strategies available for meaningful data interpretation.

### Rewiring of microglia gene regulation in Alzheimer’s disease (AD)

**Fig. 7:**
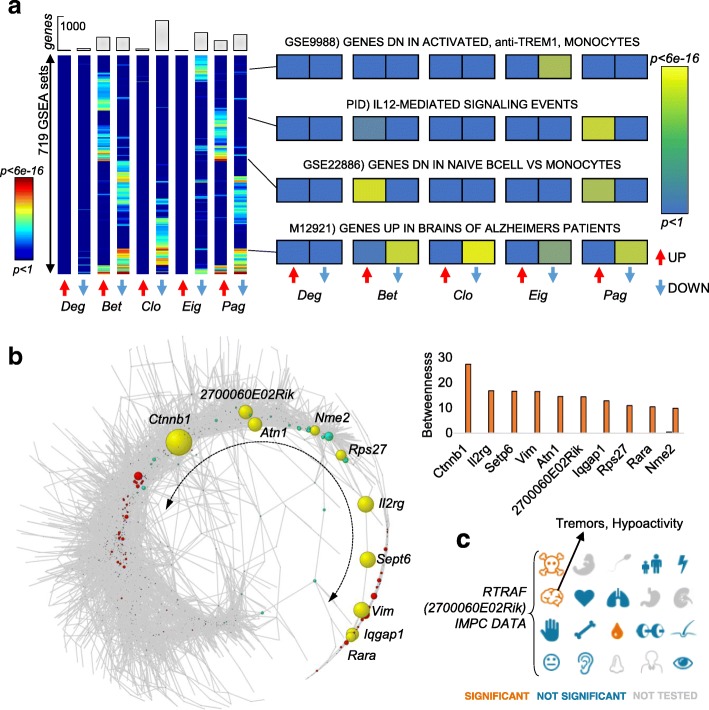
Rewiring of regulatory network in Alzheimer’s disease microglia introduces information bottlenecks. **a** MSigDB enrichments for 10 lists of genes with altered centrality in AD compared to control network (5 centralities, 719 MSigDB terms were found to be enriched with *p* < 0.05 in at least one entry). The results show that the tested centralities provide insights into different functional pathways. **b** The AD regulatory network follows a circular shape in which the nodes along the terminal tail (yellow genes) become bottlenecks, i.e., acquire increased *betweenness*. The exact increase in *betweenness* is represented in the histogram. **c** International Mouse Phenotyping Consortium data for the transcript *2700060E02Rik*, alias *Rtraf.* Homozygous *2700060E02Rik* knockout is associated with embryonic lethality prior to tooth bud stage and heterozygous 2700060E02Rik knockout is associated with tremors, hypoactivity, and increased eosinophil cell number

We further evaluated the applicability of our network-based approach in a different disease and species context with datasets of higher size and increased sparsity. To this end, we analyzed scRNA-seq data from immune cells (CD45+) derived from 5XFAD transgenic mice, a commonly used model for AD [12]. The dataset contained transcriptomes from 22,951 single cells from different disease stages (1–8 months, control, and 5XFAD) as well as *Trem2*^*+/+*^ and *Trem2*^*−/−*^ AD and control mice (*Trem2* is a key receptor that modulates immune response). The variety of conditions made the dataset particularly suitable for confirming the benefits of our unifying approach. In fact, instead of progressively fragmenting cells and conditions into stratified groups and clusters, we used all of the data as an input to generate regulatory networks for controls and AD. Overall, while the two networks were of comparable size (Table 1), we observed a general loss of connectivity in AD, which increased network sparseness and signal traveling time (shortest paths, Table 1). Consequently, centralities of several genes were different in the AD network compared to the control (Fig. 7a). Altered centralities were associated with different MSigDB enrichments, further reflecting the fact that each centrality highlights different functional aspects. For example, genes that lost influence (*eigenvalue*) significantly overlapped with genes that were dependent on *Trem1* in monocytes (*p* < 6.5 × 10^−6^). This observation is intriguing given the relevance of *Trem2* in the mediation of immune response in AD brains.

Other function–centrality associations include genes that are upregulated in AD patients (*p* < 5.0 × 10^−12^), the interleukin 12 signaling cascade (*p* < 1.1 × 10^−8^), and genes that are downregulated in naive B cells compared to monocytes (*p* < 3.5 × 10^−10^; Fig. 7a). Overall, *betweenness* showed the most dramatic changes of all centralities. In fact, the AD network was rewired into a circular shape, which in turn causes a number of genes to become information bottlenecks (Fig. 7b). Interestingly, *beta catenin 1* (*Ctnnb1*), part of the main pathway that regulates the onset and progression of AD [50], showed the largest increase in *betweenness* (from 0.0 to 27.3%, Fig. 7b) and became the main bottleneck in the AD network. Among the top 10 genes with increased *betweenness*, we also found a poorly annotated transcript *2700060E02Rik* (Fig. 7c), whose heterozygous deletion was previously associated with tremors and hypoactivity, a common symptom of AD (Mouse Phenotyping Consortium, www.mousephenotype.org).

## Discussion and conclusions

During the last decade, single-cell transcriptomics has becoming increasingly important for deconvoluting the cellular architecture of complex tissues and for classifying cells with categorizing principles. An integrated scenario, where single cells are combined to infer global regulatory networks, has not yet been comprehensively explored. There have been isolated studies using small-scale single-cell data to derive partial regulatory networks, although their reliability has been questioned [9]. Hence, it remained unclear whether single-cell datasets can be analyzed using strategies other than clustering-based phenotyping.

The main obstacles that impede network analysis of single-cell data are the technical limitations inherent to the technology and the very large data volumes. Guo and coworkers used least square fitting on expression data from 28 epithelial cells and inferred a partial regulatory network of few hundred nodes and edges [7], however, without validating it. Further, the use of least square fitting is known to perform poorly with the sparse and low complexity of single-cell data [5]. In another work [8], 92 cells were analyzed using an asynchronous Boolean approach to refine literature curated models of hematopoiesis. Boolean approaches are not easily scalable [5] and can therefore only be used to inspect reduced, specific sub-networks. Other studies applied graphical approaches not scalable to large-scale sequencing data [51–57], metrics not tailored to scRNA-seq-specific features [51, 52, 54, 55, 57, 58], or dynamic process-specific approaches [53, 54, 58, 59]. More recently, Aibar and colleagues developed the first tool designed to infer transcription factors and their target genes from single-cell data [60]. However, this tool ultimately seeks to infer one network for each cell, with consequent applications in clustering and phenotyping but not global regulatory networks.

In this work, we conceived an analytical framework for inferring large-scale regulatory networks from single-cell data. To confirm the viability of this approach, we generated a large and diverse repertoire of regulatory networks in healthy and diseased contexts. To support network interpretation, we applied tools from graph theory and validated this strategy thoroughly at multiple levels. Importantly, we showed that regulatory networks derived from single-cell data can be used to obtain novel and biologically relevant insights into the molecular architecture of complex systems and the pathophysiology of diseases. This work represents an important leap forward in the field of single-cell analysis for the reasons described below.

First, we conducted the first large-scale analysis of global regulatory networks using single cells. We processed datasets from up to 8000 single cells into networks with up to 60,000 edges and 7000 nodes, going far beyond previous studies [7, 8]. Second, we conceived a metric which consistently identified hidden correlations within the single-cell dataset. The metric was specifically tailored to single-cell data, diminishing the effect of data sparsity, confounding factors, and other technical artifacts. Thereby, it removes main obstacle to processing scRNA-seq data into regulatory networks. Third, we studied the global and local properties of networks using tools from graph theory, enabling a comprehensive characterization. Fourth, we validated our results at multiple levels. Diversified technical benchmarks with real and simulated datasets proved our method to be artifact-free, robust, reproducible, accurate, and better performing than imputation-based approaches. Fifth, we validated inferred correlations between transcription regulators and target genes via experimental signatures of perturbed biological systems. In line with previous evidence, the inferred networks were scale-free [27–29]. The centrality of genes was also validated using external experimental datasets of essential genes (OGEE database), supporting their biological relevance. Further, we validated the functionality of organ-specific central genes in their respective tissue contexts (GO enrichment). Lastly, we found that genes with altered centrality in T2D and AD strongly overlap with previous known disease mechanisms. *Sixth*, we compared the results from the regulatory network approach with those from conventional DE analysis. Notably, we found that networks repeatedly disclosed latent variation and features that were invisible to standard analysis. Moreover, we showed that gene centrality analysis was able to work in *synergy* with differential expression analysis to provide an unbiased, quantitative ranking of biological importance from dysregulated genes. To our knowledge, this is a unique strategy for deducing a data-driven biological ranking without the need to incorporate external information (e.g., GO or MSigDB) or personal knowledge. *Seventh*, we have completed the first single-cell, network-driven analysis of diseased samples. Here, graph-based tools allowed us to enhance our understanding of their molecular pathology. Our results suggest that different diseases might affect different gene centralities, as observed in Alzheimer’s disease, which primarily affected *betweenness*. This raises the possibility of different measures of centrality being sensitive to different forms of pathophysiological alterations, i.e., regulatory alterations.

In general, given its integrated rather than classifying use of single cells, we propose that the network approach is particularly well-suited for complex experimental designs with multiple confounding factors. For example, a case–control design with biased patient selection (e.g., sex and age) will inevitably result in composition biases in single-cell dataset. Disentangling such confounding from disease-related effects is a challenging task without a straightforward solution. We showed that, although greatly affecting DE analysis and clustering, such biases do not impact on gene-to-gene correlations and single-cell-derived gene regulatory networks.

In summary, we have shown that regulatory network approaches can be applied to large-scale single-cell datasets and can be used to maximize the biologically relevant information obtained. Testing consistency across multiple networks will allow us to determine the completeness of the captured regulatory interactions, and this should be the primary future task.

## Methods

### Inferring gene expression correlations and regulatory networks from scRNA-seq data

Single-cell sequencing is characterized by a series of technical limitations that generate artifacts, such as drop-out events, irregular sequencing depth, and low library complexity. First, drop-out events represent expressed genes that are undetected by scRNA-seq for technical reasons, resulting in zero values in the expression count matrix. These events make single-cell datasets considerably sparser than bulk RNA-seq datasets. Drop-out events are perhaps the most important factor affecting the performance of correlation methods, such as *Spearman* or *Pearson*, applied directly to expression count data. Second, irregular sequencing depth is caused by the uneven (non-normalized) loading of single-cell libraries into the sequencing reaction. Consequently, we observe large fluctuations in sequencing depth between cells, which can only partially be addressed by data normalization. Third, single-cell data present a reduced dynamic range of expression values, which is a further challenge for the performance of correlation methods. In fact, as it is not possible to entirely remove the effects of read distribution biases, traditional correlation coefficients have suboptimal performance with this data type. Since these technical artifacts concur to mask correlations when using expression counts, we envisaged that a change of variable would greatly improve the performance of the correlation methods, thereby allowing us to infer the regulatory networks. To this end, we devised the following steps:

1. *Data pre-processing.* Datasets were analyzed using the *bigSCale* framework [13], which handles the noise and sparsity of scRNA-seq data using an accurate numerical model of noise. The framework includes modules for differential expression analysis and unsupervised cell clustering. All datasets were processed using *bigSCale* under default parameters, with the exception of parameters regulating the granularity of clusters. *BigSCale* was set to the highest granularity (i.e., recursive clustering) in order to produce the highest number of clusters, the rationale being to segregate cell sub-types and subtle cell states, so as to improve the resolution and quality of inferred correlations.
2. *Measuring correlations in the Z-score space.* After clustering the cells to the highest feasible granularity, we used *bigSCale* to run an iterative differential expression (DE) analysis between all pairs of clusters. For *x* clusters, this results in a total of *x* × (*x* − 1)/2 unique comparisons, each yielding a *Z*-score for each gene that indicates the likelihood of an expression change between two clusters. This allows us to compute correlations between genes using *Z*-scores instead of expression values. For correlation analysis, we used *Pearson*, *Spearman*, and *Cosine* coefficients. We also tested the mutual information to detect non-linear correlations. However, in the *Z*-score space, this resulted in an excessive number of false positives. Specifically, mutual information repeatedly identified significant dependencies for which one of the two variables was linearly independent of the other (slope = 0). Nevertheless, linear correlations in the *Z*-score space can also reflect non-linear correlations in the original expression space. Hence, we chose to rely exclusively on a solid measure of linear correlation in the *Z*-score space via *Pearson*, *Spearman*, and *Cosine* coefficients. The final correlation for each pair of genes was computed as the lowest (worst) between *Pearson* and *Cosine* (*Spearman* is used in a later stage as a further control).
3. *Building a regulatory network.* In the next step, we retained significant correlations to define the edges of the regulatory network. Notably, the distribution of correlations is influenced by biological and technical factors. For example, increased cell numbers or sequencing depth results in a higher number of significant correlations. Consequently, to compare regulatory networks inferred from different datasets, we must first adjust for technical factors, for which we used an adaptive rather than fixed correlation threshold. Specifically, the inferred networks were built by retaining the top 0.1% correlations. Using this relative correlation threshold prevents technical factors from producing artificial differences when comparing different networks. Although relative thresholds could result in the inclusion of non-significant correlations (e.g., *ρ* = 0.4), we did not observe such events in any of the inferred networks, with most adaptive thresholds set between *ρ*_thresh_ = 0.9 and *ρ*_thresh_ = 0.99. The lowest (worst) adaptive threshold was *ρ*_thresh_ = 0.84 for the AD network, which is still significant. *Spearman* correlation is used as a further control to discard weak correlations. Specifically, final correlations for which |*ρ*_Spearman_| < |*ρ*_thresh_ − 0.15| were considered null.

In a final step, the undirected network is polished to retain only the edges that represent actual regulatory links. To this end, we utilized GO annotations (version 24/03/2017) to extract putative regulators of transcription (GO:0010468 “regulation of gene expression”). We discarded from the network edges representing pairs of genes of which neither was annotated as “regulator of gene expression,” as we considered these to be spurious co-expression links. Alternatively, more specific GO terms could be used for network polishing (e.g., GO:0006355 “regulation of transcription, DNA-templated” or GO:000370 “DNA-binding transcription factor activity”). However, we opted for a broader term so as to include in our networks all possible regulatory layers, including indirect signaling events. We refer to this step as GO sub-setting.

### Computing the *Z*-scores

Differential expression (DE) between clusters of cells yields a *Z*-score over which correlations are computed. DE is based on the methods previously described in [13] with two main additions.

Briefly, we generate a numerical model of the noise affecting a given single-cell dataset. Cells featuring highly similar transcriptomes are considered as biological replicas and are grouped together. Next, the expression variation within groups is used as an estimator of noise. Eventually, a *p* value is assigned to each gene, representing the likelihood of a change of expression from one biological replicate to another. This model is then generalized to compute differentially expressed genes between any given pair of cells. When identifying DE between two groups (i.e., two clusters), each cell of one group is compared to each of the cells of the other group, resulting in a total of *n*1 × *n*2 comparison, where *n* is the number of cells of each group. For each gene, the *n*1 × *n*2 log10 transformed *p* values (derived from the probabilistic model and signed to represent up- or downregulation) are summed into a total raw score. Genes up/downregulated in one group compared to the other will cumulate high (positive or negative) total raw scores. The raw score is next adjusted for the total number of comparison and for the within-group variability, which is estimated by running a DE analysis between randomly reshuffled cells in a way that cells of the same group are compared.

The first modification is adding an independent test of *Z*-scores using the Mann–Whitney *U* test/Wilcoxon rank-sum test, which was shown to be very effective on single-cell data, especially with high cell numbers [61]. For every gene, the *Z*-scores generated by the two tests (numerical model and Wilcoxon) are eventually joined in a final *Z*-score (the module of the two-dimensional vector whose dimensions are the two *Z*-scores): $\sqrt{Z_{\mathrm{num}.\mathrm{model}}^2+{Z}_{\mathrm{Wilcoxon}}^2}$. This merging rewards reproducibility that is genes with high *Z*-scores in both methods will have a higher final *Z*-score. The two tests work in synergy, filling each other’s weaknesses. For example, Wilcoxon *Z*-scores are bounded by the size of the groups: small groups will yield limited *Z*-scores, no matter how strongly the genes are differentially expressed. *Z*-scores computed with the numerical model are not limited by group sizes. On the other end, Wilcoxon is more accurate when the group sizes are large (> 500–1000 cells).

Secondly, we modified the process in which the raw score is adjusted for the total number of comparison. The rationale for this adjustment is to take into account that genes with sparser expression will produce smaller scores compared to genes expressed in high frequency. We changed from a log scale to a linear scale in the number of comparisons, allowing a far better resolution and sensitivity, especially in the range of the medium to highly expressed genes.

### Recursive clustering

The recursive clustering is the core process in our approach to identify and segregate the maximum possible amount of biologically informative cell clusters (corresponding to cells with a specific phenotype). The recursive clustering is an evolution of the previous clustering approach used in bigSCale [13], in which (1) all pairwise cell distances were computed over a set of highly variable genes (genes presenting a high degree of variation across the dataset) and (2) based on these distances cells were hierarchically clustered (Ward’s linkage).

Cutting the hierarchical tree very low (i.e., towards the leaves) would generate many small clusters. However, these clusters could lack biological relevance, because they are all created from the same initial set of highly variable genes, which is predominantly describing only the major cell types and not the sub-types or subtle cell states. For this reason, it is not suitable to cluster datasets beyond the levels of main clusters (i.e., cell types) with just one set of highly variable genes. To solve this issue, we devised a recursive clustering approach in which each cluster is further re-clustered into sub-clusters upon calculation of its specific set of highly variable genes. This is recursively repeated (each output cluster becomes the input for a further clustering) until there is no more meaningful separation.

This recursive clustering is supported by an algorithm performing, at each step, an unsupervised decision of the optimal number of clusters. The algorithm is a variation of the well-known elbow methods, modified to partition the cells in few, major clusters and to avoid over-fragmentation. We seek to avoid over-fragmentation to make the safest possible use of the highly variable genes. Briefly, the tree is cut at intervals of 10th quantiles (10th, 20th, ... 90th) and whenever an increase in the number of clusters is detected twice in a row (from example, cutting at 30th increases the number of clusters compared to 20th, and cutting at 40th further increases compared to 30th) the tree is cut at the level just before the clusters started to increase (in the previous example, 20th). This rule effectively manages to automatically divide the cells into biologically informative clusters.

Recursive clustering terminates when none of the obtained clusters can be sub-clustered in a meaningful way. Whether or not a cluster can be meaningfully sub-clustered is decided upon two elements: (1) a fixed parameter representing the lowest possible partition size (for example, set to 50 cells for datasets with less than 5000 cells) or (2) the hypothetical (unsupervised) cutting depth to which the cluster would be sub-clustered. Thus, small groups of cells can be further clustered only if they are very heterogeneous. The unsupervised cutting depth is used as a proxy for heterogeneity; the higher the cutting depth (i.e., the more we cut the tree down to its leaves), the more heterogeneity. If a given group of cells is heterogeneous enough with respect to its size (large groups of cells are clustered anyway), then it is further clustered.

### Confounding variables

Comparing healthy and type 2 diabetes (T2D) cells in DE reveals an extremely high number of differentially expressed genes. Specifically, 6716 genes are differentially expressed with *p* < 0.001. A portion of these DE genes is likely caused by confounding factors (sex, age, weight). For example, *PPP1CB* is a gene expressed approximately 14 times higher in male than in female patients, irrespectively of the disease. The same gene appears also upregulated in diabetes (*p* < 3.9e−80), simply because healthy cells have a larger amount of male cells (healthy 80% male, T2D 48% male). Consistently, the entire male signature (approximately 700 genes) is upregulated in the diabetes dataset. Also, age and weight and other unknown confounding variables generate further biases in the same way. Disentangling confounding effects from disease-related effects in single-cell patient data is not straightforward. As for the present example, the groups will inevitably present unbalanced composition, affecting DE analysis. In addition, gene-level variability (the mean and standard deviation) is very different for DE genes affected by confounding factors. Our GRN approach is stably inferring correlations, also across unbalanced sample cohorts, as long as the confounding factors are present in both groups. The correlations are stable, and only functional shifts within expression values (change of phenotype) caused by an internal rewiring of the regulatory network are detected.

### Measuring node centrality

The centrality of a node (gene) is used to quantify its importance in a network (in our case a gene regulatory network). There are different metrics to measure node centrality. Here, we used *degree*, *betweenness*, *closeness*, *pagerank*, and *eigenvalue*. All these centralities were calculated with the package igraph 1.2.2.

*Degree* is the most basic measure of centrality: it is measured as the number of edges afferent to a given node. Our inferred networks are undirected; therefore, we do not distinguish between in-degree (incoming edges) and out-degree (outgoing edges). *Betweenness* is a centrality based on shortest paths. It is calculated by enumerating all shortest paths of a network and by quantifying the number of times each node falls in a shortest path. Genes with high *betweenness* act as bridges in the signaling cascades of the network. More specifically, given that all our inferred networks showed a modular structure (Table 1), it is likely that genes with high *betweenness* serve as bridges between different modules of the network. *Closeness* centrality measures the mean distance from a node to all other nodes (by using shortest paths) of a network. Genes with high *closeness* are located in a middle, central position in the network and have therefore quick access to influence or detect the expression of any other gene. Both *betweenness* and *closeness* scale with the size of the network. In this manuscript, we always used their normalized values to avoid biases based on the network size. *Pagerank* centrality results from a random walk of the network. In simple terms, this centrality is proportional to the average time spent at a given node during all random walks. If we consider the genes as the aliases of the web pages for which *pagerank* was initially conceived, then genes with high *pagerank* can be seen as “popular” genes. *Eigenvalue* centrality uses the eigenvector corresponding to the largest eigenvalue of the graph adjacency matrix. *Pagerank* and *eigenvalue* are very similar. In fact, *pagerank* is a variation of an eigenvector-based problem. Both *pagerank* and *eigenvalue* centralities exploit the notion that not all edges are equal. In particular, edges coming from nodes with higher degree are more important than edges coming from nodes with low degree. One of the main differences between *pagerank* and *eigenvalue* is that the first includes an additional term, called damping factor, which simulates the behavior of an imaginary web-surfer who will not continue clicking indefinitely, but he/she would rather continue clicking with a certain probability. This probability is represented by the damping factor (typically 0.85).

### Network densities and GO sub-setting

The large changes observed in the network densities (Table 1) seem to be in contrast with the functioning of our adaptive correlation. In fact, the adaptive correlation threshold initially assigns the same (relative) amount of edges, hence density, to each network.

In the next steps, the networks are first cleaned by removing isolated nodes (those with zero neighbors) and isolated components (sub-networks disconnected from the main one and smaller than 1% of total network size), which leads to a decrease in density depending on the network structure. However, the subsequent GO sub-setting is probably the major driver in the reduction of network density. GO sub-setting removes all the edges not linking to at least one “regulator of transcription.” In turn, GO sub-setting triggers a second passage of edge removal, in which the nodes and components becoming isolated after GO sub-setting are removed.

GO sub-setting creates larger losses of density when genes, which are not “regulators of transcription,” initially attracted a large number of correlations. Considering that the abundance of “regulators of transcription” is stable across networks (Table 1), it means that having more or less correlations driven by non-regulators of transcription is a functional, biological feature of the tissue. Tissues with signaling cascades reaching the regulator of transcription after passing through multiple other genes are more likely to end up with lower network density. This is because the GO sub-setting aims to remove all intermediate actors that are not directly involved in the regulation of transcription.

### Validation of network edges with external datasets

Our inferred regulatory links represent putative events of the transcriptional regulation on target gene(s). We chose to also include indirect regulation events that do not imply the direct binding of a transcription factor to the promoter of the target gene(s). By filtering the edges using the broad GO term “regulators of transcription,” we included all possible regulatory layers, including transcription co-factors, epigenetic mechanisms, regulation of RNA stability/degradation, and signaling cascades. Consequently, neighboring genes (genes connected by an edge) are likely to belong to a common pathway and should be similarly affected when the system is perturbed. MSigDB contains an extensive collection of experimental signatures associated with perturbation of biological systems, which we used to independently validate each edge in our networks.

Specifically, we used collections C4 (computational gene sets), C6 (oncogenic gene sets), and C7 (immunologic gene sets) all of which defined form experimental data.

To detect significant enrichment of co-occurrences, we applied Fisher’s exact test. Edges with significant *p* values imply that the related genes are activated/deactivated together in experimentally perturbed systems significantly more often than expected by chance.

The distribution of edge-wise fold enrichment (i.e., how often the edges translate into co-occurrences in MSigDB signatures) was biased towards positive values for all mouse organs tested, indicating an overall simultaneous modulation of neighboring genes (Fig. 3e, f). Co-regulation was further supported by significant *p* values (e.g., Fig. 3g, Additional file 1: Figure S3a), especially when considering edges with higher numbers of associated MSigDB signatures (small gene sets are less likely to yield significant *p* values in the Fisher exact test). Notably, we inferred organ-specific regulations, whereas the MSigDB signatures are collected from a highly heterogeneous set of biological sources. Inevitably, some of our organ-specific regulatory links will be not backed-up by MSigDB signatures, which explains why we could not validate not all individual edges in our networks.

For all the GO (version 24/03/2017) and MSigDB (version v6.0) enrichment analyses, we used hypergeometric distribution with *Bonferroni* correction.

### Validation of network hubs with gene essentiality

To elucidate whether the hubs in our networks represent essential regulatory factors, we took advantage of the Online GEne Essentiality (OGEE) database. This database provides an unbiased, comprehensive catalogue of the essentiality of experimentally tested genes across species. In this setting, we used the *Mus musculus* dataset (available at http://ogee.medgenius.info/browse/Mus%20musculus), which lists the essentiality status for 9402 mouse genes. To quantify the essentiality of each set of hubs, we computed an essentiality score (ES), as:

$$ ES={\log}_2\frac{\frac{E_{\mathrm{hubs}}}{NE_{\mathrm{hubs}}}}{\frac{E_{\mathrm{background}}}{NE_{\mathrm{background}}}} $$

where *E*_hubs_ and *NE*_hubs_ are the number of essential and non-essential hubs, and *E*_background_ and *NE*_background_ are the number of essential and non-essential genes in the OGEE dataset, respectively.

To assess the significance of each ES, we computed the empirical probability of finding a score of the same magnitude by chance. Specifically, given a set with *N* hubs, we sampled *N* random genes from the OGEE dataset and calculated the ES. We repeated this process 10,000 times, and from the resulting distribution, we used the one-tailed *p* value as the proportion of random ES that are equal to or greater than the observed ES. After calculating one *p* value for each ES, we corrected for multiple testing by applying a Benjamini–Hochberg correction to the vector of *p* values.

### Detection of changes in centralities

We evaluated two different approaches for ranking nodes according to their change in centrality. The first approach identifies the highest absolute change in centrality, where for each node is defined as the difference in its centrality between networks A () and B (). Next, we selected the 1000 nodes with the greatest change in centrality (either positive or negative). The change in centrality was then integrated with the *p* values of the DE analysis (*bigSCale*, standard parameters) to identify genes undetected by DE (Additional file 1: Figure S8a). In an alternative approach to identify relative changes in centrality, we searched for dispersed nodes lying outside the proportional relationship between *c*_*a*_ and *c*_*b*_. We performed non-linear fitting (smoothing spline) to derive a confidence interval of the dispersion. Nodes that showed overdispersion at *p* < 0.05 were defined as having altered centrality (Additional file 1: Figure S8b). Ultimately, we did not use this analysis in the manuscript, opting for the absolute change only (first approach). This is because relative changes in centrality, as measured by overdispersion, were biased towards small changes in centrality, which were important at a relative level, but irrelevant at the absolute level.

### Organ-specific genes

The specificity of the genes in the network was quantified using their connectivity and expression. For the former, nodes were considered as specific if they were ranked in the top 20% of a given centrality measure exclusively in a particular organ. The putative function of each set of organ-specific nodes was assessed by GO enrichment analysis using the GOstats package [62].

Alternatively, the expression multiplicity of each gene in the network (the number of organs in which it is exclusively expressed) was computed as described in [6]. Briefly, we calculated a modified *Z*-score for each gene in each network, in which the difference between the mean expression of a gene in a specific organ and its median expression across organs is divided by the interquartile range (IQR) of its expression across organs. The multiplicity is obtained by counting how many organs have a *Z*-score > 2, and organ-specific genes are those with a multiplicity = 1.

## Additional files

Additional file 1: **Figure S1.** Benchmarking inferred correlations. **Figure S2.** Single-cell gene regulatory networks are scale-free. **Figure S3.** Validation of inferred networks and analysis of multiplicity. **Figure S4.** Relationship between degree and other centralities. **Figure S5.** The central genes of different metrics show marginal overlap. **Figure S6.** Relationship between gene centrality and biological essentiality. **Figure S7.** Monotone behavior of healthy and diseased pancreatic tissue. **Figure S8.** Detection of genes showing changes in centrality. (PDF 1880 kb)

Additional file 2: MSigDB enrichments for gene sets with altered centrality. (XLSX 14300 kb)
