# Supplementary material for: Single-cell transcriptomics unveils gene regulatory network plasticity
Source: Genome Biol. 2019 Jun 4;20:110. doi: 10.1186/s13059-019-1713-4 (PMC6547541; doi:10.1186/s13059-019-1713-4)
Supplement: Supplementary file 1 — Figure S1. Benchmarking inferred correlations. Figure S2. Single-cell gene regulatory networks are scale-free. Figure S3. Validation of inferred networks and analysis of multiplicity. Figure S4. Relationship between degree and other centralities. Figure S5. The central genes of different metrics show marginal overlap. Figure S6. Relationship between gene centrality and biological essentiality. Figure S7. Monotone behavior of healthy and diseased pancreatic tissue. Figure S8. Detection of genes showing changes in centrality. (PDF 1880 kb) [file 13059_2019_1713_MOESM1_ESM.pdf]

Additional file 1

Supplementary figures 1-8

Supplementary Figure 1

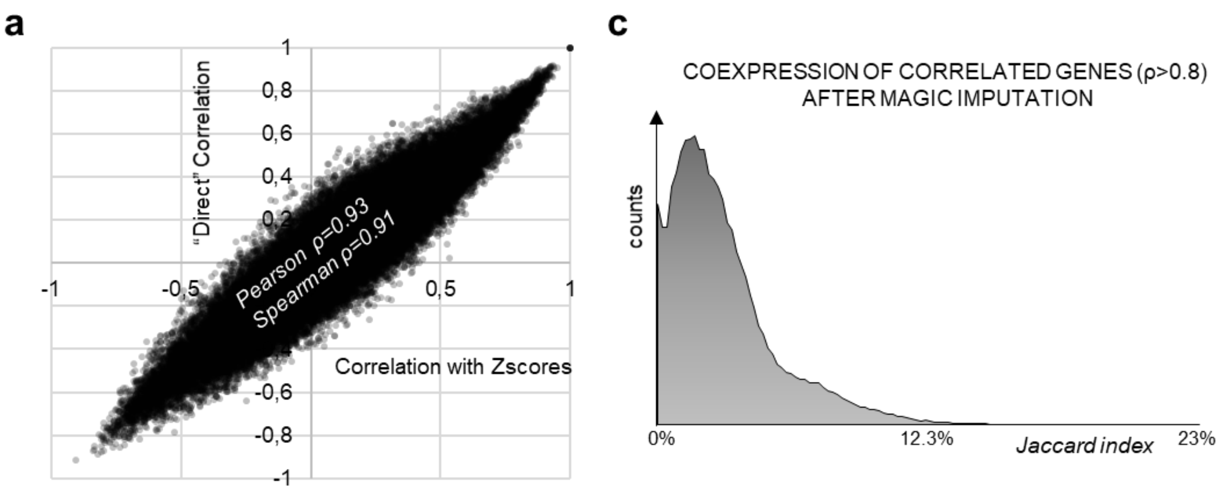

b

Iteration	Correlations ρ>0.8
1	1128
2	321
3	704
4	1234
5	826
**AVERAGE**	**842**
**REAL**	**1349102**
**FDR**	**0.062%**

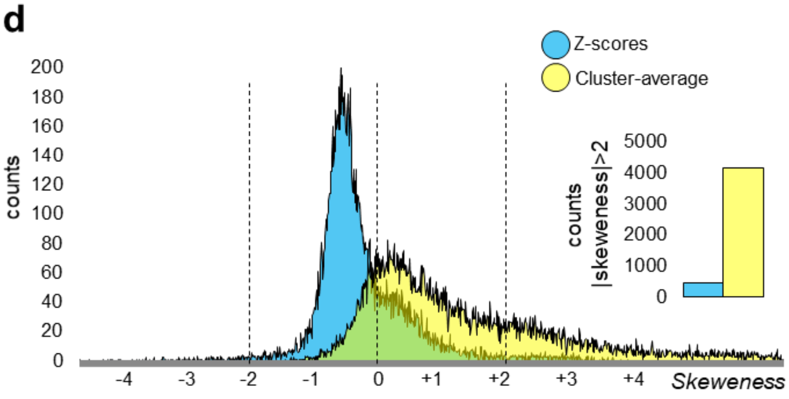

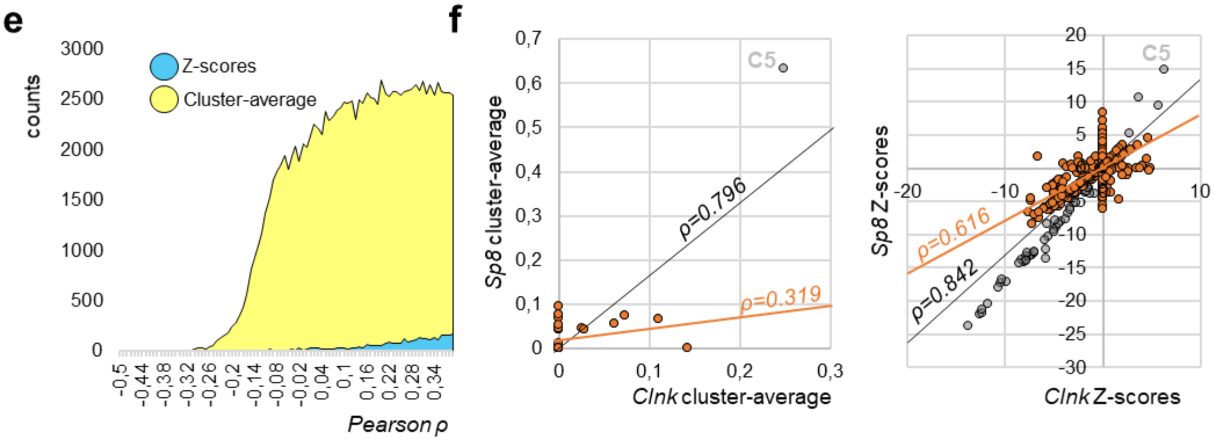

Fig. S1 Benchmarking inferred correlations

a Comparing Zscore-based correlations with correlations computed over average cluster expression (3005 brain cells from Zeisel 2015). In the second scenario, every cluster yields one observation and the total number of observations is equal to the total number of clusters (48, as determined by the recursive clustering in an unsupervised manner). The two approaches show a remarkably high agreement of values. Specifically, we found 606,732 correlations with *Pearson* ρ>0.8 when computed "directly" or with Z-scores. This magnitude of intersection is highly significant (p<1e-1000, too low to be computed exactly with double precision variables). Furthermore, we compared the overall similarity of the 121,609,810 (=19972*19971/2 genes) correlations computed either with Z-scores or the "direct" method (Supplementary figure 2a). We found a highly significant monotone (*Spearman*=0.91) and more specifically linear correlation (*Pearson*=0.93) between the two approaches. As expected, the Z-score approach is more sensitive than the "direct" method, as it finds roughly 2.1-fold more correlations with ρ>0.8 (1,349,102 vs 642,788). Of note, the 642,788 "direct" correlations already greatly exceed the 210 correlations found using the cell-wise normalized counts (Fig. 2c, ρ>0.8). An improvement was expected, as averaging the gene expression per cluster is already diminishing the effects of drop-out events, which are the major cause of the poor performance of cell-wise correlation analysis. Together, the results indicate that Z-score correlations are in line with "direct" correlations.

b Random cluster assignment is used as a null model to quantify FDR. For each random cluster assignment, the table shows the number of significant correlations found. The number is then divided by the amount or real correlations to calculate the FDR.

c Co-expression plot for the genes correlated (ρ>0.8) after MAGIC imputation. Average co-expression is very low (2.58%). The same dataset processed with our tool yielded an average co-expression of 31.99% (ρ>0.8). The difference in correlations is larger when rising the cutoff to ρ>0.8: MAGIC yields a 2.6% average correlation, our approach 43.58%.

d Gene-wise skewness (computed as m_3/s^3, where m_3 is the sample moment of order 3 and s the standard deviation) of the cluster-average values and of Z-scores values, 3005 brain cells from Zeisel 2015. We first segregated the cells in 48 clusters using our recursive clustering. Next, for each gene (15596 genes) we computed cluster-average expression values and relative skewness. This was compared with the skewness of the 1128 values (Z-scores) yield by the iterative differential expressions (48*47/2) between pairs of clusters. Z-scores have a peak centered around zero and present only 455 genes with a skewness higher than +/-2, which is almost 10-fold reduction compared to the 4152 genes with high skewness in cluster-average data. This occurs because typically only one or few clusters will be positive for a given gene, leaving

the majority of cluster-average values floating around zero and giving little to none contribution in the determination of the correlations.

e Changes in correlations caused by the loss of one cluster. We sought to prove that cluster-average correlations, being driven by few outliers, cannot make an efficient use of the information of all clusters. To test this hypothesis, we dropped one cluster at the time and quantified the impact on the correlations using either cluster-average or Z-scores. Specifically, we counted how many correlations drop from $\rho>0.8$ to $\rho<0.6$ after discarding clusters one by one. As expected, the total amount of losses in correlations using cluster-average is higher than using Z-scores (125648 vs 3108, 40x increase).

f Correlation between the genes *Clnk* and *Sp8* before (gray line) and after (orange line) the removal of cluster 5 from the analysis. Using cluster-average approach a correlation of 0.796 is inferred when using all the 48 clusters. However, this correlation is largely dependent on the sole cluster number 5 (C5), as dropping its value results in the loss of the detected correlation (orange dots, $\rho=0.319$). This occurs because cluster-average is not sensitive and accurate enough to use the information of the remaining clusters to detect the correlation. This does not happen with our Z-score processing, in which the loss in correlation is more contained ($\rho=0.616$).

Supplementary Figure 2

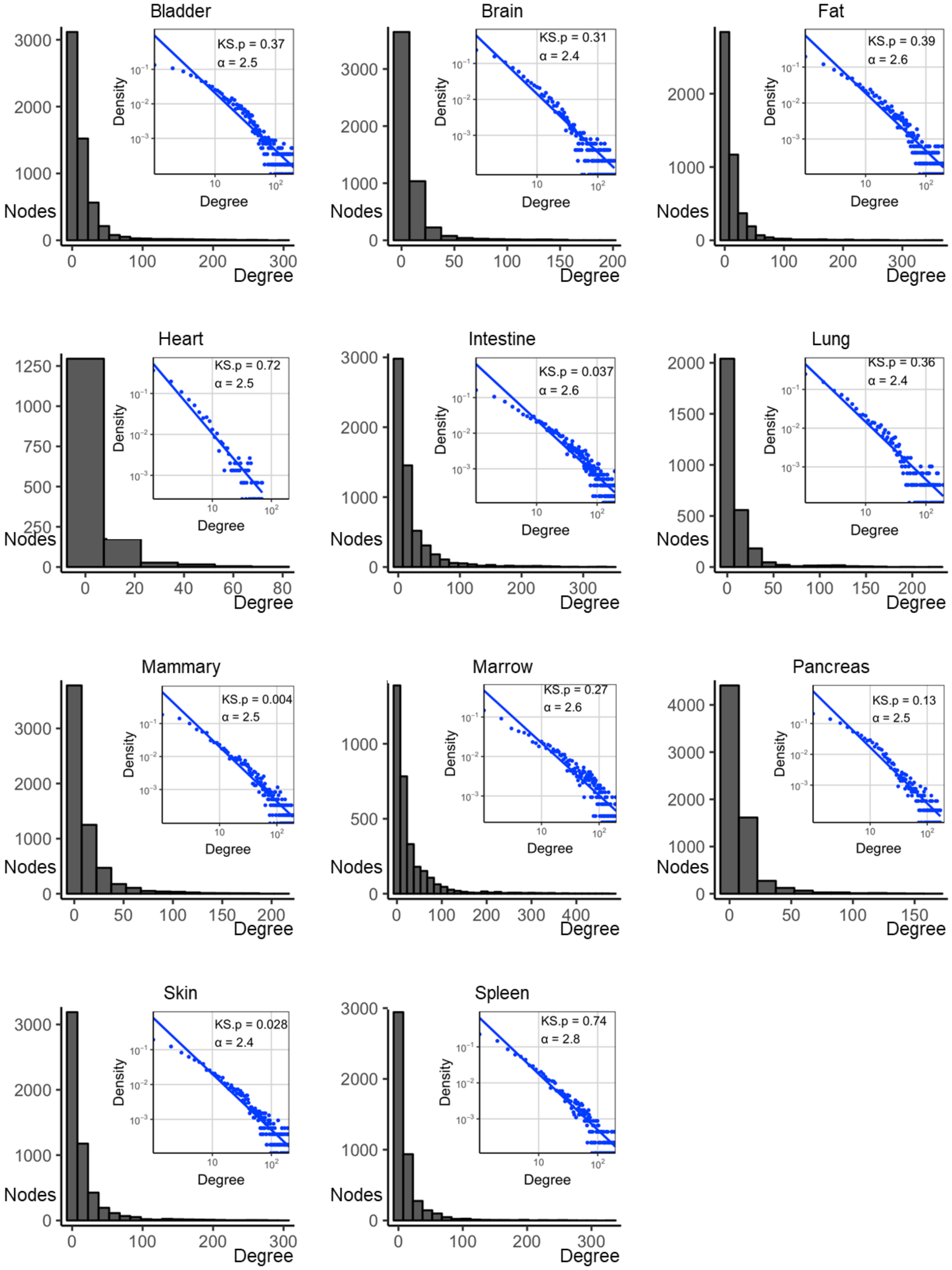

Fig. S2 Single-cell gene regulatory networks are scale-free.

The degree distribution of the single-cell gene regulatory networks derived for 11 organs shown in linear (histogram) and logarithmic scale (scatter plot). Each distribution was fitted to a power-law distribution, and the p-value of the Kolmogorov-Smirnov test (KS.p) and the degree exponent of the power-law (alpha) are shown for each network. All networks are scale-free ($p>0.01$) apart from the mammary gland which slightly deviates from the exact scale-free distribution ($p<0.004$).

Supplementary Figure 3

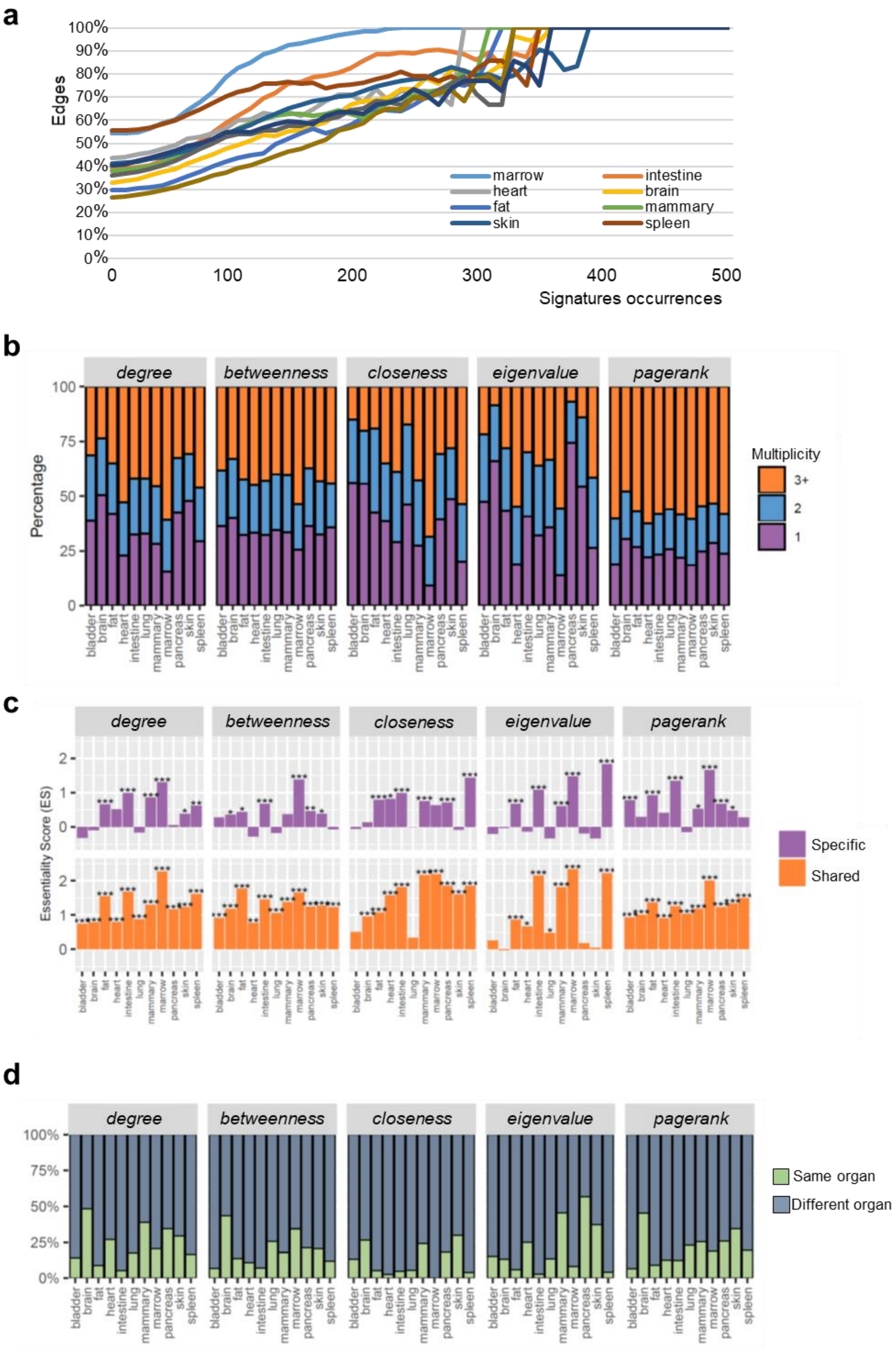

Fig. S3 Validation of inferred networks and analysis of multiplicity.

a Percentage of validated edges validated with $p<0.05$ (y-axis) filtered by the number of GSEA occurrences (x-axis) for each organ. Increasing occurrences correspond to higher percentages of edges validated by significant p-values ($p<0.05$). **b** The central genes (top 20%) of each organ classified by their multiplicity for all tested centrality measures. Multiplicity=1 means that they are central only in that organ, whereas multiplicity=2(3+) means that they are central also in additional organs (total of 2, or 3 or more, organs). **c** Comparison of ES score of organ-specific central genes (multiplicity=1) against shared central genes (multiplicity 3+). The latter have higher biological essentiality (* $p<0.05$, ** $p< 0.01$, *** $p<0.005$, random permutations, see methods). **d** Analysis of genes which are i) central in at least one organ and ii) up-regulated in one organ compared to others. Intriguingly, most of the genes central in a given organ are expressed to a significantly higher extent ($p<0.05$) in a different organ. The brain has the highest amount of genes which are central and more expressed (compare to other organs) at the same time.

Supplementary Figure 4

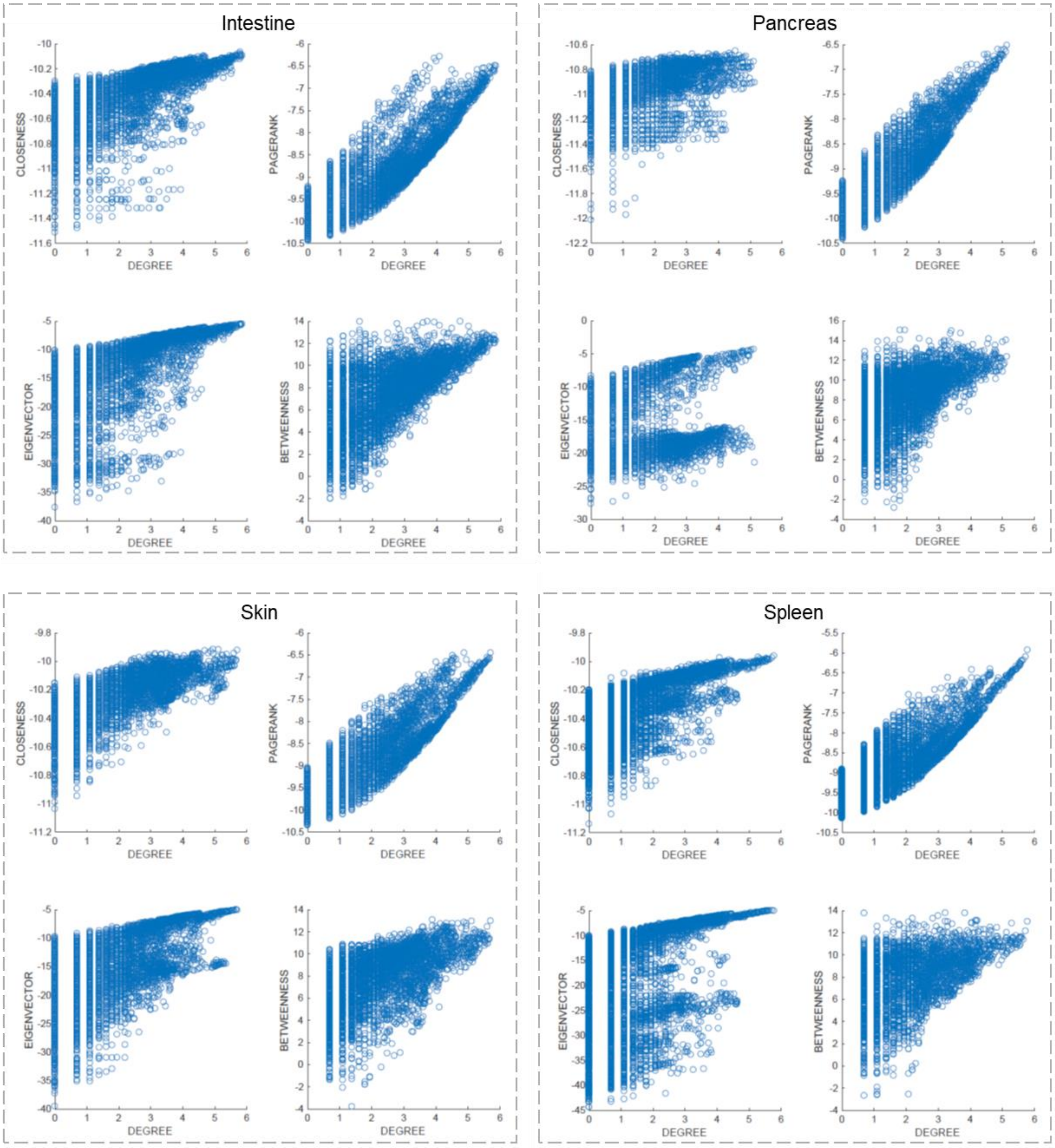

Fig S4. Relationship between degree and other centralities.

Scatter plots between *degree* (log-scale) and the other centralities. *Degree* is perhaps the most simple and direct measure of centrality. Nodes with high degree have many connections and are therefore more likely to be central also in the other metrics. In line, *degree* and the other metrics show a general positive correlation, as shown in the examples of the intestine, the pancreas, the skin and the spleen. However, the other metrics are able to capture types of node importance (i.e. centrality) which the *degree* cannot. This is shown by the sparseness and/or multi-modality of several distributions, such as for example *degree* and *pagerank* in the intestine, where nodes with the same degree can present widely different *pagerank* centrality.

Supplementary Figure 5

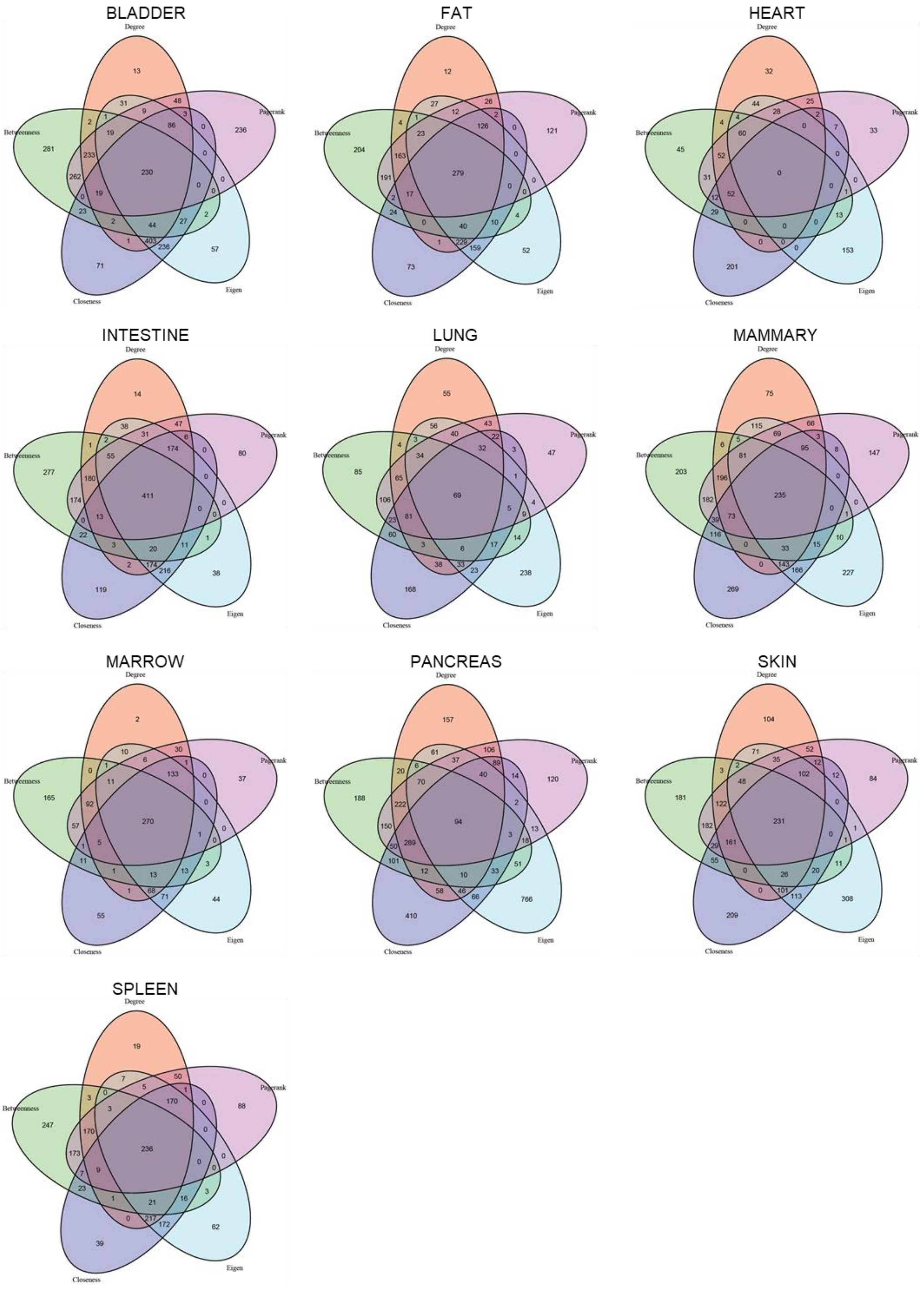

Fig S5. The central genes of different metrics show marginal overlap.

Venn diagrams intersecting the genes central (top 20%) in different measures.

Supplementary Figure 6

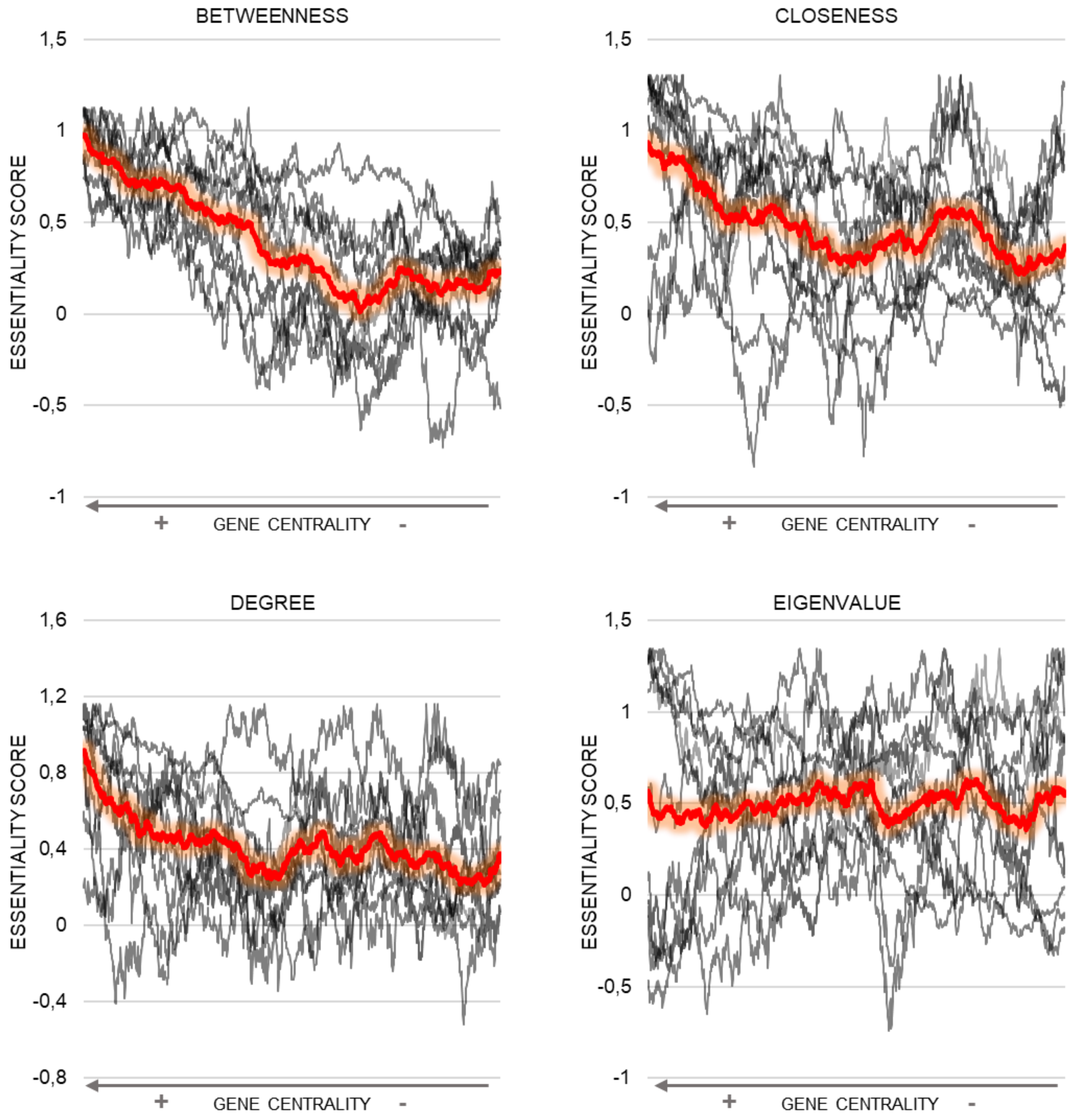

Fig S6. Relationship between gene centrality and biological essentiality.

Genes sorted according to their centrality. The top central genes (left side of the x-axis) in *betweenness*, *closeness* and *degree* show the highest biological essentiality (ES score, OGEE database, see methods). *Eigenvalue* has an unstable performance (working for some organs and not for others), depending on the structure of the network. *Closeness* does not perform well on disconnected graphs.

Supplementary Figure 7

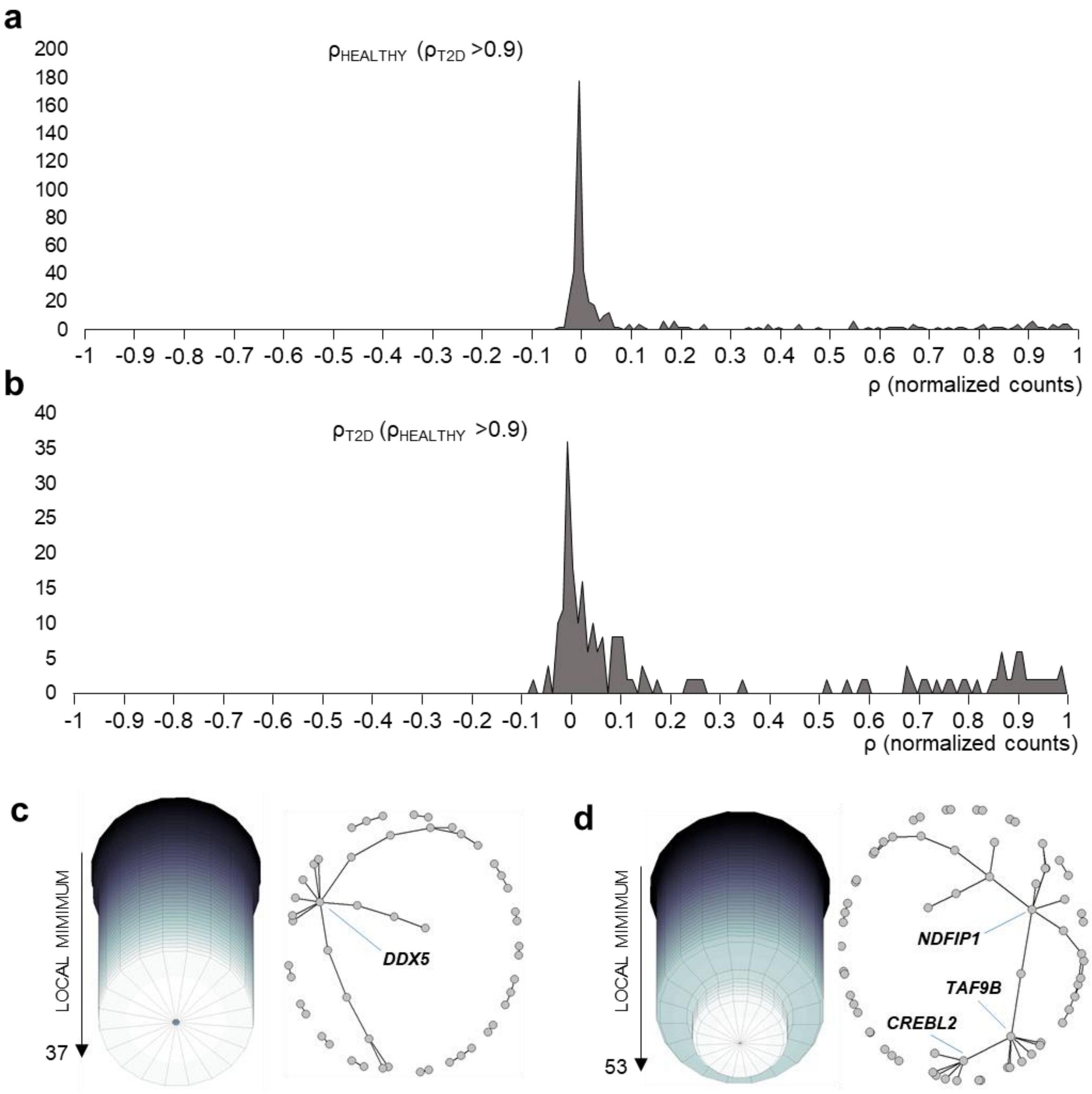

Fig S7. Monotone behavior of healthy and diseased pancreatic tissue

a,b Persons correlation inferred directly from normalized expression counts are few and not reproducible. The correlations (correlated gene pairs) significant in T2D ($\rho>0.9$) are mostly scattered around zero in the healthy dataset (a) and vice versa (b), indicating poor reproducibility as opposed to the results of our approach (Fig. 6c). In addition, the significant correlations are few compared to what found with our approach (Healthy, 258 $\rho>0.9$ with expression counts, 235947$\rho>0.9$ with our approach, T2D, 484 $\rho>0.9$ with expression counts, 335909 $\rho>0.9$ with our approach). This is in line with what we found in the other datasets (Fig. 2c). **c,d** Dynamical behavior is perhaps the most important aspect of biological modeling, and describes how systems respond to input or perturbation. Biological regulatory networks have been suggested to display nearly monotone behaviors [35], [36], i.e. the prevalence of predictable, bounded trajectories over "chaotic", oscillatory behaviors. Generally, abundant negative signs (negative correlations between genes) favor non-monotone behaviors. A social network is an intuitive parallel of this, in that unfriendly relationships (negative signs) increase social tension and decrease social monotonicity [37]. We considered it interesting to assess whether our scRNA-seq-derived regulatory networks preserve the near-to-monotone behavior previously found in manually curated networks [35], [38]. The T2D regulatory network possessed more negative edges than the healthy counterpart, suggesting that diabetes causes an increase in chaotic signaling in the pancreas. In a way, the monotonicity can be seen as a proxy to test the homeostasis of a biological system. In general, biological systems seek to maintain homeostasis to achieve optimal functioning. The monotonicity, given its bounded trajectories favouring steady states, is one aspect of such preference of biological systems for homeostasis. In turn, given that homeostatic imbalance (inability to maintain homeostasis) may lead to a disease condition, we hypothesize that certain disease conditions might display a decrease of monotonicity. Computing the distance to monotonicity (how many negative edges must be removed to achieve monotonicity) of large networks is a complex, NP-hard (non-deterministic polynomial-time) problem, for which there are only approximate solutions. We used a greedy heuristic based on gauge transformations and previously shown to be the most accurate solution for large networks [36]. As expected from the number of negative edges, the T2D network was less monotone than the healthy network (53/52035=0.101% compared to 37/48549=0.076% residual negative edges, respectively). The nodes carrying these residual negative signs have potentially chaotic effects in the dynamic behavior of the network. Herein, we identified these potentially chaotic transcription regulators in healthy (DDX5) and T2D (NDFIP1, TAF9B and CREBL2) networks (Fig. 5f,g). However, both networks were extremely close to monotone, in comparison to previously reported regulatory

networks (Yeast 3.8%, E.coli 11.2% residual negative edges) [36]. Hence, the decrease in monotonicity in T2D was not sufficient to suggest that diabetes induces “chaotic” signaling in the pancreas. Nevertheless, this approach illustrates the potential for going beyond clustering-based phenotype analysis of single cells, allowing us to infer advanced system properties, such as dynamical behaviors of organs in health and disease. While we did not observe increased “chaotic” signaling in T2D, is it still intriguing to speculate that this is the case in other diseases.

Degree of the monotonicity (residual negative edges) was 37 for control (**a**) and 53 for T2D (**b**). Nodes carrying residual negative edges are highlighted in the networks plots.

Supplementary Figure 8

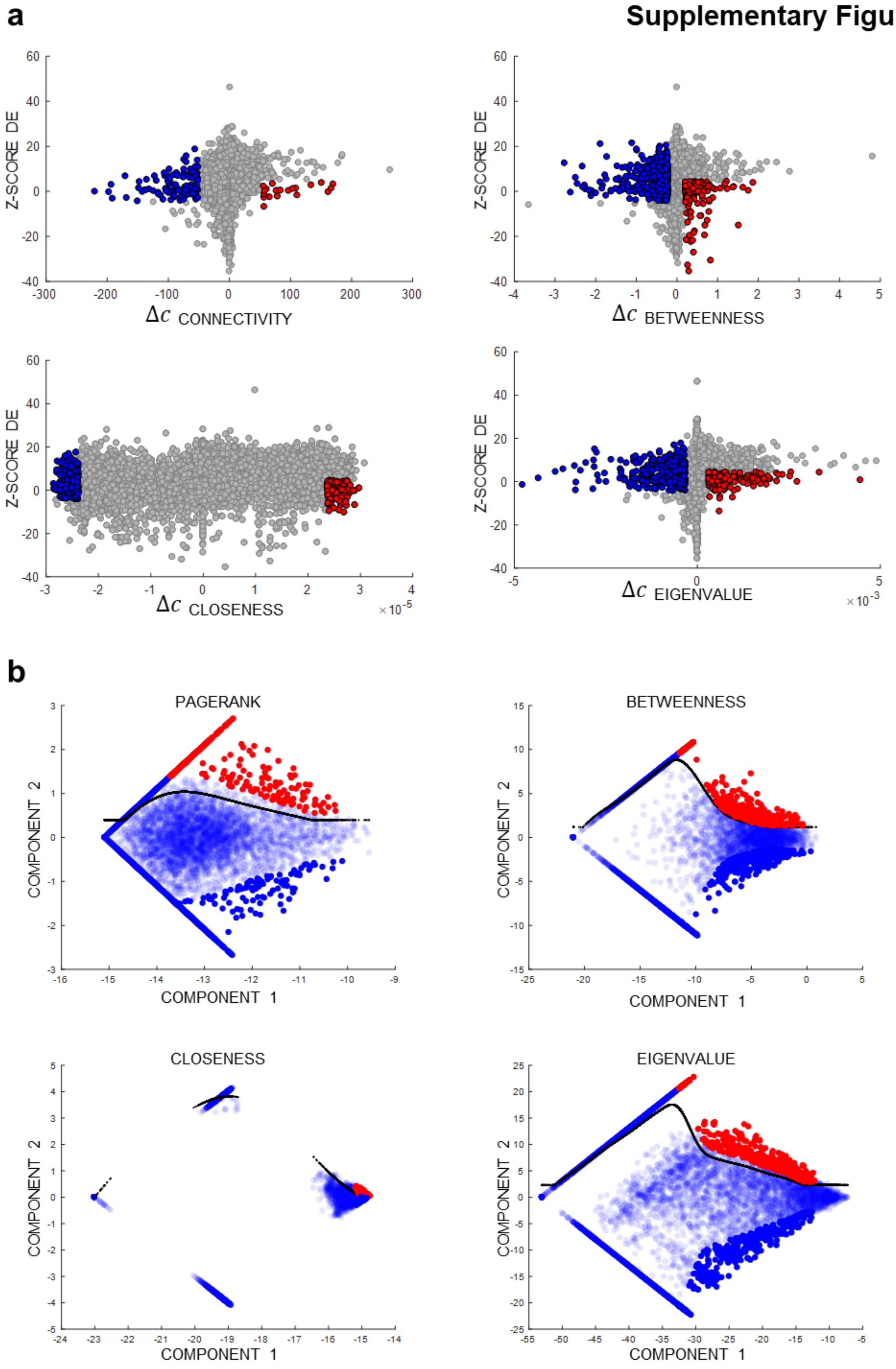

Fig S8. Detection of genes showing changes in centrality.

a Change in expression (Z-score, y-axes) compared to the absolute change in the five centralities.

b Adaptive empirical fitting for the detection of relative changes of centralities.
